# Discovering the potential of *S. clavuligerus* for bioactive compound production: cross-talk between the chromosome and the pSCL4 megaplasmid

**DOI:** 10.1186/s12864-017-4289-y

**Published:** 2017-11-25

**Authors:** Rubén Álvarez-Álvarez, Yolanda Martínez-Burgo, Antonio Rodríguez-García, Paloma Liras

**Affiliations:** 1 Microbiology Section, Faculty of Biological and Environmental Sciences, University of León, León, Spain; 2 Institute of Biotechnology of León, INBIOTEC, León, Spain

**Keywords:** *Streptomyces clavuligerus*, pSCL4 megaplasmid, Plasmid evolution, Transcriptome analysis, Clavulanic acid, Secondary metabolites

## Abstract

**Background:**

*Streptomyces clavuligerus* ATCC 27064, the industrial producer of the β-lactamase inhibitor clavulanic acid, carries 49 putative secondary metabolite gene clusters. These secondary metabolite gene clusters are distributed between its linear chromosome and the 1.8 Mb-plasmid pSCL4, a rich reservoir of bioactive compound gene clusters.

**Results:**

The transcriptome and metabolome of *S. clavuligerus* ATCC 27064 and the pSCL4^−^ derived strain, *S. clavuligerus* pSCL4^−^, were analysed. Construction of the *S. clavuligerus* pSCL4^−^ strain resulted in the excision of a 303 kb stretch in the right arm of the chromosome and its translocation to pSCL4, producing a 2.1 Mb plasmid named pSCL4* .

The absence of pSCL4* results in changes in the transcription level of genes encoding regulatory proteins or proteins with various functions. Lack of pSCL4* results in upregulation of three chromosomal gene clusters for secondary metabolites (SMC), SMC18, for holomycin and N-propionylholothin biosynthesis, SMC11b for tunicamycin biosynthesis (located between SMC10 and SMC11), and SMC5. The SMC10, SMC11 and SMC6 gene clusters were downregulated, resulting in lower production of clavulanic acid, cephamycin C and desferrioxamine E, respectively. Clusters SMC8, SMC12, SMC13 and SMC19 were also downregulated. Production levels of bioactive compounds, such as alkylresorcinol or thiol-derived compounds, were affected in the plasmid-less strain.

**Conclusions:**

The excision and translocation to pSCL4 of 303 kb from the right arm of the chromosome confirms that the ends of the chromosome arms are regions of high instability and supports the hypothesis that pSCL4 might have been excised from *S. clavuligerus* chromosomal right arm end. Cysteine and methionine metabolism in *S. clavuligerus* lacking pSCL4* may differ from that of the wild type strain, given the absence of sulfur metabolism genes located either in pSCL4 or at the right end of the chromosome, which led to levels of dithiolopyrrolones (holomycin, N-propionylholothin) and acetylhomocysteine thiolactone (citiolone) higher than those of the wild type strain. *S. clavuligerus* pSCL4^−^ shows strong differences in its transcriptome and metabolome; however, the loss of 2.1 Mb DNA is dispensable in this strain.

## Background

*Streptomyces clavuligerus* ATCC 27064 is the industrial producer of clavulanic acid, a β-lactamase inhibitor widely used in combination with the semisynthetic β-lactam amoxicillin to treat various bacterial infections. Forty-nine putative secondary metabolite gene clusters (SMC) have been identified in this species [1], one of the largest number of SMCs found in any bacterium. These SMCs are distributed between its lineal chromosome, with 24 SMCs, and a rich reservoir of secondary metabolic pathways, the 1.8 Mb-plasmid pSCL4, encoding 25 SMCs. *S. clavuligerus* ATCC 27064 has three additional plasmids: pSCL1, pSCL2, and pSCL3, of 11.7, 120, and 430 kb in length, respectively [2], but SMCs have not been identified within them so far.

Most of these gene clusters for bioactive compounds are silent and/or cryptic [1]. Charusanti et al. [3] exploited adaptative laboratory evolution by co-cultivation of *S. clavuligerus* with a methicillin-resistant *Staphylococcus aureus* strain; in this way they obtained fourteen *S. clavuligerus* isolates producing holomycin, a bioactive compound whose gene cluster is silent in the wild type strain in tested laboratory conditions. Two of these strains lack the whole pSCL4, and a third one lacks the leftmost stretch of pSCL4. Also, the holomycin producer strains *S. clavuligerus oppA2::aph* [4] and *S. clavuligerus claR::aph* [5] showed a lower copy number of pSCL4 than the wild type strain, suggesting that pSCL4 might be involved in the activation of some secondary metabolite gene clusters.

To obtain a *S. clavuligerus* strain devoid of pSCL4, the *parA-parB* genes for pSCL4 segregation were deleted in the wild type strain [6]. *S. clavuligerus* pSCL4^−^ grew slightly slower than the wild type strain, but was a viable strain, indicating that the pSCL4 plasmid is dispensable [6]; likewise, in accordance with previous studies [3, 6, 7], *S. clavuligerus* pSCL4^−^ produced a large amount of holomycin.

In addition to its large potential to produce a wide variety of bioactive compounds, the megaplasmid pSCL4 carries some regulatory genes [1]. Among them are the only gene for a butyrolactone receptor in *S. clavuligerus* genome, genes for serine/threonine kinases, genes for pairs or orphan two-component regulatory systems, and for other regulatory protein families. Therefore, given the in silico cross-talk prediction between chromosomal and pSCL4-encoded genes [1], to gain more in-depth knowledge of the potential of *S. clavuligerus* in bioactive compound production, the metabolome and transcriptome of *S. clavuligerus* ATCC 27064 and the derived *S. clavuligerus* pSCL4^−^ strain were analyzed.

The rapid evolution of the array of secondary metabolites in *Streptomyces* may be due to the instability of their chromosome ends that leads to amplifications and deletions in the genome and contributes to transmision of genetic information between *Streptomyces* plasmids and the chromosome. In this sense the double recombination of a smaller plasmid with the chromosome seems to be the most plausible scenario to explain the origin of pSCL4 [1].

Our studies confirm a megaplasmid-chromosome cross-regulation and support that pSCL4 may be originated by excision from the right arm of *S. clavuligerus* chromosome as previously suggested [1].

## Results

### Insights on the evolution of pSCL4

**Fig. 1:**
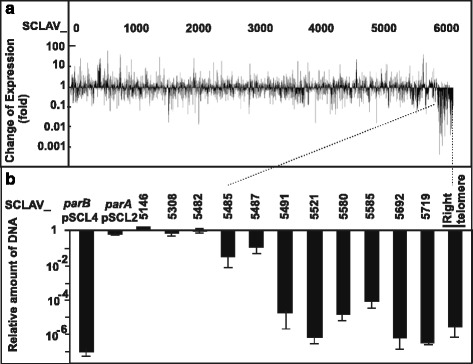
Change of gene expression level and DNA quantification of genes in *S. clavuligerus* pSCL4- in relation to wild type strain. **a** Change of expression level of genes in *S. clavuligerus* pSCL4^−^ chromosome at 46.5 h in relation to the wild type strain. The pattern of the change of gene expression level was the same at 22.5 h and 60 h (not shown). **b** qPCR of: i) pSCL4 located genes (*parB*_pSCL4_) deleted in *S. clavuligerus* pSCL4^−^, ii) genes located in plasmids different from pSCL4 (*parA*_pSCL2_), iii) genes located in the central part of the chromosome (SCLAV_5146, SCLAV_5308), and iv) genes located in the 303 kb stretch of the right arm of the chromosome (SCLAV_5482, 5485, 5487, 5491, 5521, 5580, 5585, 5692, 5719 and the right telomere (nt 6,760,214 to 6,760,380)

During the analysis of the transcriptome of both *S. clavuligerus* pSCL4^−^ and *S. clavuligerus* ATCC 27064, performed in the exponential and stationary growth phases, the genes along the chromosome in *S. clavuligerus* pSCL4^−^ were up- or downregulated in relation to the control strain; however, the coding DNA sequences (CDS) in the rightmost stretch of the right arm of the chromosome had systematically lower transcription levels in the three sampling times (Fig. 1a). These results might be due to a modulation of the transcription of these genes, located close to the right telomere, as a result of the lack of pSCL4. An alternative explanation is the absence of these genes in the pSCL4^−^strain.

To determine the origin of this downregulation, the following genes were analyzed by qPCR in both strains: i) 10 genes located in the underexpressed region of the right end of the chromosome (between SCLAV_5482 and the right telomere), ii) genes located in the central region of the chromosome (SCLAV_5146, SCLAV_5308) or located in plasmid pSCL2 (*parA*_pSCL2_), and iii) genes, such as *parB*_pSCL4,_ located in pSCL4, and therefore absent in *S. clavuligerus* pSCL4^−^ (Fig. 1b).

**Fig. 2:**
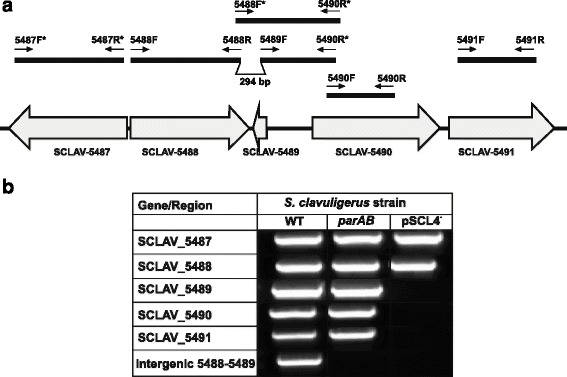
PCR-based location of genes SCLAV_5487 to SCLAV_5491 in the *Streptomyces* studied strains. **a** The genes and regions indicated were analyzed in *S. clavuligerus* ATCC 27064, *S. clavuligerus parAB::aac* and *S. clavuligerus* pSCL4^−^ using the indicated oligonucleotides. **b** Regions amplified to determine the position in which the excision occurred in *S. clavuligerus parAB::aac.* The excision occurred at the 294 nt intergenic region shown in the upper part of the figure

The qPCR analysis showed the same number of copies of SCLAV_5146, SCLAV_5308 and *parA*_pSCL2_ in *S. clavuligerus* pSCL4^−^ as in the wild type strain. As expected, in *S. clavuligerus* pSCL4^−^ amplification of *parB*_pSCL4_ was undetectable_,_ and the same occurs to the 10 genes located in the right arm of the chromosome that were analyzed (Fig. 1b). In the DNA stretch showing lower transcriptional signal level (Fig. 1a), the relative amount of DNA ranged from 10^−6^ for SCLAV _5719 to 10^−4^ for SCLAV_5585 (Fig. 1b), suggesting that genes located downstream of SCLAV_5491, including the right telomere, are not present in *S. clavuligerus* pSCL4^−^. In order to determine when the translocation occurred, strains *S. clavuligerus* ATCC 27064, *S. clavuligerus parAB::aac,* derived from the wild type strain, and *S. clavuligerus* pSCL4^−^ constructed from *S. clavuligerus parAB::aac*, were analyzed by final time PCR to test every gene between SCLAV_5487 and SCLAV_5491 (Fig. 2). While all the genes were present in *S. clavuligerus* ATCC 27064 and *S. clavuligerus parAB::aac,* genes SCLAV_5489 to SCLAV_5491 were absent in *S. clavuligerus* pSCL4^−^. This suggests that the excision occurred downstream of SCLAV_5488 (Fig. 2b); therefore, the intergenic SCLAV_5488 to SCLAV_5489 was analyzed; a qPCR amplification product was detected in the wild type strain, but not in *S. clavuligerus parAB::aac* or *S. clavuligerus* pSCL4^−^ (Fig. 2b).

The amplification of genes SCLAV_5487 to SCLAV_5491, and the lack of amplification of the SCLAV_5488 to SCLAV_5489 intergenic region in *S.clavuligerus parAB::aac* (Fig. 2b) suggests that those genes were already excised from the chromosome in this strain, and translocated to plasmid pSCL4 to form a 2.1 Mb megaplasmid named pSCL4*. The region in which the excision may take place is 294 nt in length (Fig. 2a) and may form a hairpin loop in all its length with a ΔG value of −85.98 kcal/mol (Additional file 1: Figure S1). This secondary structure might facilitate the excision of the 303 kb chromosomal DNA fragment, subsequently translocated to the megaplasmid and lost when pSCL4* is eliminated in *S. clavuligerus* pSCL4^**−**^ **.** An alternative hypothesis is that the deletion of pSCL4 *parA-parB* segregation genes forces the integration of the megaplasmid in the right arm of the chromosome to avoid its loss, but due to the large size of this plasmid, the right arm of the chromosome might have split at one of the chromosomal hot spots during replication**.** Therefore, when we refer to the lack of pSCL4 in strain *S. clavuligerus* pSCL4^**−**^ **,** the lack of the 303 kb chromosomal DNA fragment is always included.

Most of the genes in the translocated chromosomal DNA fragment encode hypothetical proteins, but others encode regulators, proteins involved in antibiotic resistance, genes with different functions and four gene clusters for secondary metabolism (SMC20 from SCLAV_5489, SMC21, SMC22 and SMC23).

Therefore, in the transcriptome studies presented below, we will consider only genes up to SCLAV_5488 since the lower expression of the final 231 CDS (SCLAV_5488 to SCLAV_5719) is due to their absence in the studied strain.

### Transcriptome analysis

To analyze possible crosstalk regulation between genes located in *S. clavuligerus* chromosome and genes in the megaplasmid pSCL4, the transcriptome of *S. clavuligerus* pSCL4^−^ [6] was compared to that *S. clavuligerus* ATCC 27064, the wild type strain. Transcriptome analysis based on microarrays showed that, as expected, the 1570 CDS located in pSCL4 showed a negative M_g_ value (which is proportional to the abundance of the transcript for a particular gene), in the plasmid-less strain due to the absence of the transcripts for these genes. In total, 210 chromosomal genes were upregulated in the strain lacking pSCL4, 15 corresponding to regulatory genes and 34 included in secondary metabolites gene clusters. In addition, 335 chromosomal genes were downregulated, including 30 genes encoding regulatory proteins and 25 genes located in secondary metabolites gene clusters. Furthermore,

by metabolomics analysis, differences in secondary metabolites produced between both strains were identified.

### Production of antibiotics

The production of clavulanic acid, cephamycin C and holomycin was assessed in *S. clavuligerus* ATCC 27064 and *S. clavuligerus* pSCL4^−^ grown in SA media. The lack of pSCL4 resulted in lower cephamycin C production and a decrease in clavulanic acid production (about 50% along the fermentation) (Additional file 1: Figure S2). Holomycin, not detectable in the wild type strain, was produced by the pSCL4^−^mutant at high levels (885 μg/ mg DNA at 70.5 h), as described previously [6].

**Fig. 3:**
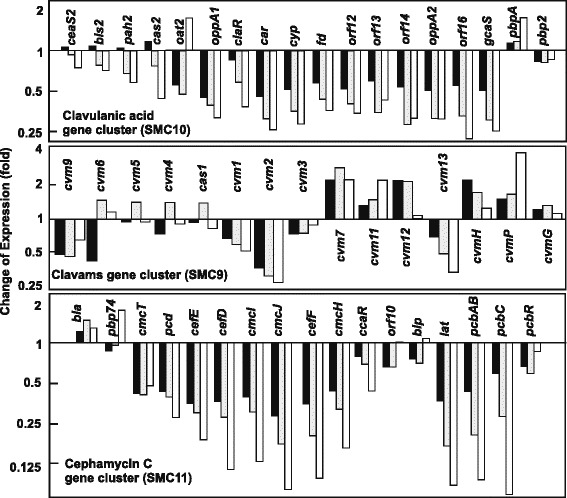
Effect of the lack of pSCL4 on β-lactam biosynthesis gene clusters expression. Transcription level of genes of SMC10, SMC9 and SMC11 antibiotic gene clusters. Expression of the genes for the biosynthesis of clavulanic acid (SMC10), clavams 5*S* (SMC9), and cephamycin C (SMC11) in *S. clavuligerus* pSCL4^−^ is compared with those in *S. clavuligerus* ATCC 27064 (taken as 1.0). Bars represent the change of expression (fold) (base-2 logarithmic scale) at early exponential phase (black bars), exponential phase (grey bars) and stationary phase growth (white bars). The name of the genes is indicated over or below the bars

Concomitantly, all the genes for clavulanic acid production (cluster SMC10) were downregulated (Fig. 3, upper panel). The effect was weaker for genes of the early steps (*ceaS2, bls2, pah2, cas2*) than for genes of the late steps of the pathway. This global downregulation of the gene cluster may be consequence of *claR* low expression level (2.9-fold decrease), as described by Martínez-Burgo et al. [5]. Lack of pSCL4 affected differently the genes in the clavams gene cluster (SMC9) (Fig. 3, central panel). Upregulation of *cvm7, cvm11, cvm12, cvmH* and *cvmP* was observed at all the sampling times but *cvm9, cvm1, cvm2* and *cvm13* were downregulated. The decrease in cephamycin C production observed in *S. clavuligerus* pSCL4, fits well with the lower expression of the genes for the biosynthetic early (*pcbAB, pcbC*) and late steps (*cefD, cefE, cefF, cmcH, cmcI, cmcJ*) of the cephamycin C pathway (cluster SMC11, Fig. 3 lower panel).

All genes from *hlmA* to *hlmI*, for holomycin biosynthesis (cluster SMC18), were strongly upregulated, although the effect of the lack of pSCL4 on *hlmK*, *hlmL* and *hlmM* was lower (not shown), as previously described [7].

### Lack of pSCL4: effect on expression level of chromosome genes

*Secondary metabolites gene clusters.* Gene-clusters for secondary metabolites (SMC) in *S. clavuligerus* have been detected using the antiSMASH prediction method [1, 8, 9]. Transcription levels of genes for the secondary metabolites biosynthesis were heterogeneous. Chromosomal gene clusters SMC20 to SMC23 were not present in *S. clavuligerus* pSCL4^−^ and expression of clusters SMC14 to SMC17, for the formation of two NRPS, a type II PKS and a phytoene/squalene type of compound, respectively, were not affected by the lack of plasmid pSCL4.

**Fig. 4:**
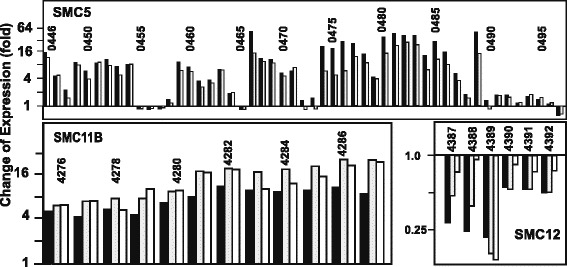
Effects of the lack of pSCL4 on putative gene clusters for secondary metabolites biosynthesis. Change of expression (fold) of the upregulated SMC5 (upper panel) and SMC11b (lower left panel), clusters, and the downregulated SMC12 (lower right panel) gene clusters of *S. clavuligerus* pSCL4^−^ in comparison with those in *S. clavuligerus* ATCC 17064 (taken as 1.0). Only the exponential phase samples are shown for SCM5. Bars represent the change of expression (fold) (base-2 logarithmic scale) at early exponential phase (black bars), exponential phase (grey bars) and stationary phase growth (white bars). The gene SCLAV_0465 has no probe in the microarrays. The SCLAV_ number of the genes is indicated above the bars

Tunicamycin and holomycin have been reported to be produced by *S. clavuligerus* [10]. Clusters SMC5, SMC11b and SMC18 for a type I PKS, for tunicamycin and for holomycin, respectively, showed all or most of their genes upregulated in the pSCL4-less strain (Table 1), as shown in detail for the tunicamycin and the SMC5 clusters (Fig. 4, lower left and upper panels).

**Table 1:**
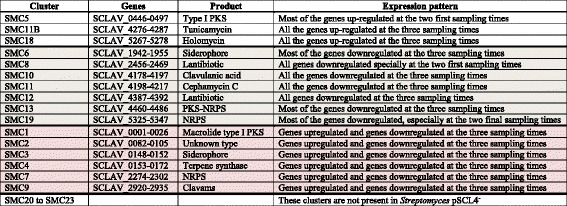
Expression patterns in genes located in secondary metabolites gene clusters

Clusters SMC6, SMC8, SMC10, SMC11, SMC12, SMC13 and SMC19 encoding compounds with very different chemical structure (lantibiotics, β-lactams, PKS-NRPS, NRPS) or acting as siderophores [11], showed all or most of their genes downregulated in *S. clavuligerus* pSCL4^−^ (Table 1, shadowed in grey) as also shown with detail for the clavulanic acid or the cephamycin C gene clusters (Fig. 3, upper and lower panels) and for genes of the SMC12 cluster (Fig. 4, lower right panel). Other clusters SMC1, SMC2, SMC3, SMC4, SMC7, and SMC9 showed gene- and time-dependent up- or downregulation (Table 1, shadowed in dark grey).

The specific molecular structure of many of the compounds formed by the SMCs of *S. clavuligerus* is unknown and only the type of enzymes encoded by some genes allows to associate a type of chemical structure to these SMCs, but no correlation was detected between the transcription levels of the genes and the chemical structure of the compound produced by the clusters.

*(ii) Regulators.* Forty five chromosome regulatory genes were up- or downregulated in *S. clavuligerus* pSCL4^−^ at least 2-fold at all the sampling times (Table 2). SCLAV_2377, encoding the sigma factor SigE is one of the upregulated genes. Located in the operon *sigE-cseA-cseB-cseC,* this gene is part of a signal transduction pathway that allows *S. coelicolor* to sense and respond to changes in the integrity of its cell envelope [12]. Genes encoding the other three components of this pathway (CseA, a negative regulator; CseB, a response regulator; and CseC, a sensor histidine protein kinase) were also upregulated. A strong effect was also observed on the *ahpDC*-*oxyR* operon, which is activated by OxyR, as defense response to oxidative stress [13]. These genes were strongly upregulated at stationary growth phase (Table 2). SCLAV_4082 (*rpoE*), orthologous to *S. coelicolor* SigR [14], was upregulated at exponential growth phase. RpoE controls a regulon of 113 genes involved in oxidative stress control. Sixty four genes of this regulon were slightly upregulated (equal to or higher than 1.5-fold in at least one of the three sampling times) as described for *S. clavuligerus* Δ*claR* [5]. The antisigma RsrA coding gene was also slightly upregulated at exponential growth phase.

**Table 2:** Genes encoding regulatory proteins^a^ which are up- or down-regulated in *S. clavuligerus* pSCL4^-^

Gene	Product	M_c_			BH-corrected *P*-value			Fold change		
		22.5 h	46.5 h	60 h	22.5 h	46.5 h	60 h	22.5 h	46.5 h	60 h
Up-regulated										
SCLAV_2377	ECF-subfamily RNA polymerase sigma factor	2.42	1.91	1.76	1.80E^-05^	1.43E^-04^	2.61E^-04^	5.36	3.77	3.39
SCLAV_2378	Lipoprotein cseA	1.30	0.83	1.23	1.55E^-05^	1.08E^-03^	8.98E^-06^	2.46	1.77	2.35
SCLAV_2379	Transcriptional regulatory protein cseB	1.34	1.08	1.71	1.35E^-03^	6.52E^-03^	5.56E^-05^	2.54	2.11	3.26
SCLAV_2380	Sensor protein	1.60	1.22	2.19	6.07E^-04^	5.17E^-03^	9.20E^-06^	3.04	2.33	4.56
SCLAV_3934	Alkyl hydroperoxide reductase ahpD	0.61	1.24	3.66	3.34E^-01^	2.15E^-02^	2.27E^-07^	1.53	2.37	12.63
SCLAV_3935	Alkyl hydroperoxide reductase	0.57	1.27	3.90	3.35E^-01^	1.29E^-02^	3.82E^-08^	1.49	2.41	14.89
SCLAV_3936	Putative LysR-family transcriptional regulator	1.11	1.48	2.06	1.47E^-02^	1.09E^-03^	2.09E^-05^	2.16	2.79	4.17
SCLAV_4082	RNA polymerase sigma factor RpoE	1.23	1.64	0.66	2.61E^-03^	1.06E^-04^	8.26E^-02^	2.35	3.11	1.58
SCLAV_4083	Putative anti-sigma factor	1.06	1.30	0.29	4.63E^-03^	5.29E^-04^	4.56E^-01^	2.08	2.46	1.22
Down-regulated										
SCLAV_0691	RNA polymerase sigma factor	^−^1.35	^−^1.62	^−^1.21	2.66E^-03^	3.19E^-04^	3.78E^-03^	2.55	3.08	2.32
SCLAV_2573	WhiB-family transcriptional regulator	^−^2.74	^−^3.47	^−^4.47	6.05E^-04^	3.12E^-05^	9.92E^-07^	6.68	11.11	22.16
SCLAV_2833	Putative serine/threonine kinase anti-sigma factor	^−^1.16	^−^1.70	^−^2.54	4.77E^-02^	2.92E^-03^	3.22E^-05^	2.24	3.24	5.81
SCLAV_3047	Predicted transcriptional regulator	^−^1.43	^−^2.74	^−^2.45	2.53E^-02^	5.41E^-05^	1.40E^-04^	2.69	6.70	5.47
SCLAV_3146	TetR-family transcriptional regulator	^−^1.04	^−^1.20	^−^1.55	5.19E^-03^	1.16E^-03^	5.07E^-05^	2.06	2.29	2.93
SCLAV_3422	Transcriptional regulator	^−^1.80	^−^2.65	^−^3.79	3.07E^-02^	1.37E^-03^	2.01E^-05^	3.49	6.27	13.82
SCLAV_3814	ECF-subfamily RNA polymerase sigma factor	^−^1.27	^−^1.08	^−^1.71	5.63E^-03^	1.38E^-02^	1.94E^-04^	2.42	2.11	3.27
SCLAV_4929	Regulatory protein	^−^1.71	^−^1.98	^−^3.90	7.07E^-03^	1.64E^-03^	1.49E^-06^	3.28	3.94	14.92

^a^Only some of the most affected genes are shown

The lack of pSCL4 resulted in thirty regulatory genes downregulated with a minimal 2-fold difference at all the sampling times (Table 2). A slight downregulation was observed on SCLAV_0691 and SCLAV_3814 genes, that encode *S. coelicolor* SigB and SigQ sigma-like factors, respectively, involved in morphological differentiation [15, 16] and on SCLAV_3146, encoding a transcriptional factor similar to *S. coelicolor* AtrA [17, 18]. Strongly downregulated was SCLAV_2573, for a transcriptional regulator similar to *S. coelicolor* WblA [19]. Two additional transcriptional regulators of unknown function shown in Table 2 were also downregulated.

**Fig. 5:**
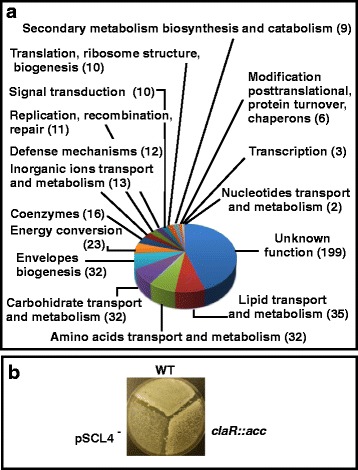
Effects of the lack of pSCL4 on genes with different functions. **a** The pie chart shows miscellaneous genes affected in *S. clavuligerus* pSCL4^−^ classified by their role in the cell according with the COG database. The number of genes affected is indicated in parenthesis. **b** Effect of the lack of pSCL4^−^ and *claR* in the formation of aerial mycelium and spores. *S. clavuligerus* ATCC 27064, *S. clavuligerus* pSCL4^−^ and *S. clavuligerus* Δ*claR*::*aac* were grown in plates of ME medium during 9 days. Note the extracellular complementation on the formation of aerial mycelium between the wild type strain, or *S. clavuligerus* pSCL4^−^, and *S. clavuligerus* Δ*claR*::*aac*, and the lack of effect of *S. clavuligerus* ATCC 27064 on *S. clavuligerus* pSCL4^−^

*(iii) Genes with diverse functions.* In addition to the genes already mentioned, 446 genes encoding proteins with different functions in the cell, were affected in the pSCL4^−^ strain, 161 upregulated and 285 downregulated (Fig. 5a). The most upregulated genes were SCLAV_0783 to SCLAV_0785, orthologous to SCO1557 to SCO1559, which encode a methionine transport system [20]. Expression of some of these genes will be discussed below in relation to the metabolites produced by *S. clavuligerus.*

*(iv) Differentiation. S. clavuligerus* pSCL4^−^ shows a very poor or null aerial mycelium or spores formation [6]. Concomitantly, several genes involved in differentiation were affected by the lack of pSCL4 (Additional file 1: Table S1). Seven genes involved in cell wall and membrane biogenesis were upregulated including SCLAV_5204, which encodes a *SsgA-*like protein (SALP) involved in peptidoglycan synthesis and the thickening of the spore cell-wall [21]. Also upregulated was SCLAV_1824, which encodes a small mechanosensitive channel involved in hypoosmotic stress protection [22] and, as previously mentioned, SCLAV_2573, which encodes a WhiB-like transcriptional regulator affecting morphological differentiation [19].

Twenty six genes related to environmental adaptation and differentiation, were downregulated in the pSCL4^−^ strain, including SCLAV_5177 and the block of genes from SCLAV_5181 to SCLAV_5192, downregulated in the three sampling times. These genes are orthologous to *S. coelicolor mce* operon, likely involved in survival in natural environment [23].

Aerial mycelium and sporulation of *S. clavuligerus* Δ*claR,* which is defective in *amfS* expression [5], are extracellularly complemented by cross-feeding both by the wild type strain and by *S. clavuligerus* pSCL4^−^ (Fig. 5b). This indicates that *S. clavuligerus* pSCL4^−^ forms the SapB peptide encoded by *amfS* [24], as occurs with the wild type strain. The lack of extracellular complementation between the wild type strain and *S. clavuligerus* pSCL4^−^ (Fig. 5b) may be explained by the downregulation of *atrA*, *wblA*, *sigB*, *sigQ* and/or upregulation of *sigE* in the pSCL4-minus strain.

### Validation of the transcriptome data

The transcriptome data previously showed was validated with 46.5 h RNA samples using RT-qPCR. Twelve genes were validated, including genes for the biosynthesis of clavulanic acid (*oppA2*), cephamycin C (*pcbC*), holomycin (*hlmA*); several genes encoding regulatory proteins (SCLAV_3410, SCLAV_4464, SCLAV_4650, SCLAV_5308), as well as five genes for miscellaneous proteins (Additional file 1: Figure S3A). The RT-qPCR values consistently confirmed the transcriptome data obtained in the microarrays. A Pearson’s correlation coefficient of 0.99 between the data from the two techniques was obtained (Additional file 1: Figure S3B).

### Metabolomic analyses: Effect of the lack of pSCL4 on secondary metabolites production

**Fig. 6:**
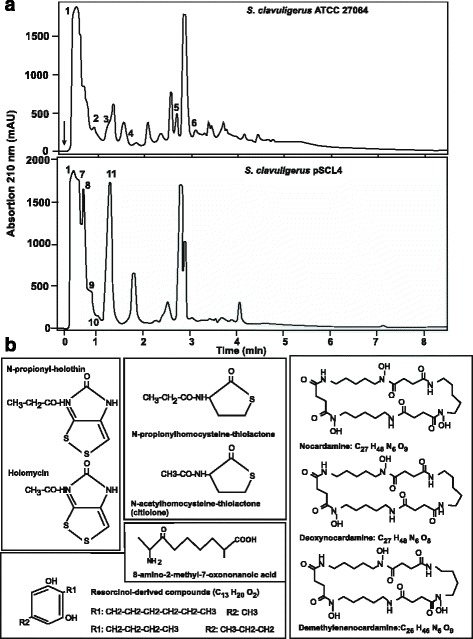
Comparative HPLC analysis between *S. clavuligerus* ATCC 27064 and *S. clavuligerus* pSCL4^−^ **. a** Components determined in *S. clavuligerus* ATCC 27064 extracts (upper pannel) and *S. clavuligerus* pSCL4^−^ extract (lower panel). 1:MOPS; 2: demethylenenocardamine and deoxynocardamine; 3: Nocardamine; 4: 8-amino-2-methyl-7-oxononanoic acid, 5: 2-hexyl-5-methyl-1,3-benzenediol (resorcinol) and 2-butyl-5-propyl-resorcinol, 6: 2-hexyl-5-methyl-1,3-benzenediol and 2-butyl-5-propyl-resorcinol, 1: MOPS, 7:citiolone, 8: N-propanoyl-3-aminodihydro-2(3H)-thiophenone (N-propionyl-homocysteine thiolactone); 9:holomycin, 10:nocardamine, 11: N-propionylholothin. **b** Chemical structure of the above indicated compounds

The ability of *S. clavuligerus* pSCL4^−^ to produce secondary metabolites is lower than that of the wild type strain since 25 clusters for secondary metabolism are located in pSCL4 [1] and 3 clusters in the translocated 3’end region of the chromosome, all of which are deleted in *S. clavuligerus* pSCL4^−^. Indeed, concentrated broth extracts of *S. clavuligerus* pSCL4^−^ and *S. clavuligerus* ATCC 27064 analyzed by HPLC, showed notable differences in the 210 nm absorption pattern (Fig. 6a).

*S. clavuligerus* ATCC 27064 and *S. clavuligerus* pSCL4^−^ were grown in SA medium up to stationary phase as indicated above. For each strain, one 100 ml sample containing broth and mycelium was analyzed. Both strains produced compounds with structure of cyclic peptides, identical to those with siderophore activity (Fig. 6b). A peak of desferrioxamine E (nocardamine) was present in the broth extract of both strains (peaks 3 and 10, Fig. 6a) at different concentrations. In addition, the wild type strain showed small amounts of demethylenenocardamine and deoxynocardamine (peak 2, Fig. 6a) which were not detectable in the mutant. These desferrioxamine-related compounds have been previously described in a *Streptomyces* sp. isolated from a marine sponge [25].

Several organic acids present in extracts of the wild type strain, were not detectable in the *S. clavuligerus* pSCL4^−^ strain. These compounds derived from linear organic acids, i.e., 8-amino-2-methyl-7-oxononanoic acid (peak 4), or from resorcinol (1,3-benzenediol), i.e., 2-hexyl-5-methyl-resorcinol (with the same molecular mass as 2-butyl-5-propyl-resorcinol) that appears as mixtures in peaks 5 and 6 (Fig. 6b).

The most characteristic peaks found in *S. clavuligerus* pSCL4^−^ extracts were compounds containing sulfur, which are not detectable in the wild type strain. The aromatic sulfur compounds, N-acetylhomocysteine thiolactone (citiolone) and the potentially toxic N-propionyl-3-aminodihydro-2(3H)-thiophenone, were identified in peaks 7 and 8, respectively (Fig. 6b). Both compounds have relatively similar structures and their biochemical origin is probably the lactone derived from homocysteine (2-amino-4-sulfanyl-butanoic acid) with N-acetyl or N-propionyl substitutions.

The other sulfur compounds found in *S. clavuligerus* pSCL4^−^ belong to the dithiolopyrrolone family (Fig. 6b), which corresponds well with the high production of holomycin (peak 9) and the high expression level of the holomycin biosynthesis genes in this strain (not shown). Surprisingly, the largest peak for a dithiolopyrrolone in our chromatogram corresponds to N-propionylholothin (peak 11), (with molecular weight identical to that of thiolutin). N-propionylholothin has been already described in an uncharacterized *Streptomyces* strain producing cephamycin C [26], supporting that peaks 11 corresponds to N-propionylholothin rather than to thiolutin, that has never been detected in *S. clavuligerus*. The MS and UV absortion spectra of all the compounds differentially produced by the wild type strain and the pSCL4^−^ mutant are shown in Additional file 1: Figure S4.

As far as we know, with the exception of the dithiolopyrrolones, all the other compounds found by HPLC-MS in this work, i.e., nocardamine and their derived analogs, homocysteine-derived lactones, linear organic acids or resorcinol-derived compounds, have never been previously described in *S. clavuligerus.*

## Discussion

Medema et al. [1] proposed that pSCL4 might have evolved by recombination between a small plasmid and the *S. clavuligerus* chromosome. After excision, the megaplasmid would carry a large fragment of the chromosome. Excision of pSCL4 from the chromosome occurred mainly at a specific site to give a 1.8 Mb pSCL4 plasmid [1, 27], but different excision sites in the chromosome might exist. In the process of deleting the *parAB*_**pSCL4**_ genes [6] we enriched and amplified a clone in which a chromosomal DNA fragment (303 kb, 231 CDS) was excised from the right arm of the chromosome and translocated to pSCL4 (1.8 Mb) resulting in the 2.1 Mb pSCL4* megaplasmid. The claim that all the genes in the chromosome are present in the megaplasmid-less *S. clavuligerus* strain obtained by adaptive evolution [3] suggest that these authors obtained a 1.8 Mb pSCL4^−^ in which no translocations ocurred.

The translocation of these chromosomal genes to the megaplasmid in *S. clavuligerus parAB::aac* is confirmed by their loss, as shown by PCR and by their low transcriptional signal level, in *S. clavuligerus* pSCL4^−^. Our finding confirms that the ends of the chromosome arms are regions of high instability [28] and supports the hypothesis that pSCL4 might have been excised from the chromosomal right arm end [1]. As a result of the lack of *S. clavuligerus parAB::aac* right telomere, this strain might have a circular chromosome, as described previously for other telomere-less linear *Streptomyces* genomes [29–31].

Deletion of only one regulator encoding gene, *claR*, located in the clavulanic acid cluster, produced multiple effects on the transcriptome of *S. clavuligerus* Δ*claR* [5] and affected several gene clusters for secondary metabolism in this strain. There are 132 regulatory genes located in pSCL4. It contains genes for two component systems (17), for regulators of the LysR-type (4), AraC-type (7), TetR-type (10), ArsR-type (7), MarR-type (6), SARP-type (3), XRE-type (5) and genes for different types of additional regulators, including sigma factors (6), and the only butyrolactone-receptor protein (SCLAV_p0894) in *S. clavuligerus* [32, 33]. Lack of plasmid pSCL4 has, not surprisingly, a broad and large effect. These regulators might act directly, or in cascade, on multiple genes located in secondary metabolites clusters, or encoding miscellaneous genes or regulators located in the chromosome.

In agreement with the lower clavulanic acid production in *S. clavuligerus* pSCL4^−^, genes of the SMC10 cluster (for clavulanic acid formation) are slightly downregulated. Also downregulated are the clusters SMC6 (for desferrioxamine E), SMC11, SMC12 and SMC19.

In the upregulated gene clusters is especially important the upregulation of cluster SMC18, for holomycin biosynthesis [7], which correlates well with the production of this dithiolopyrrolone in *S. clavuligerus* pSCL4^−^ cultures. Also upregulated are SMC5, and SMC11b for tunicamycin biosynthesis. These results agree with the holomycin-tunicamycin overproduction described by Kenig and Reading [10] in the *S. clavuligerus* IT1 strain. However, tunicamycin was not detected in our samples.

A rich array of different compounds was found in the broths of the wild type and the pSCL4^−^ strain cultures. Desferrioxamine E was present in the supernatants of both strains, confirming that the siderophore biosynthesis cluster is located in the chromosome. Clusters SMC3 and SMC6 are candidates to encode nocardamine biosynthesis enzymes. Both clusters, but specially SMC6, are downregulated in the pSCL4-less strain, what may agree with the absence of desferrioxamine -related demethylenenocardamine and deoxynocardamine, and the lower amount of desferrioxamine, in this strain.

Alkylresorcinols are autoregulators in some Gram-positive bacteria reported to interact with DNA and modify its structure and viscosity [34]. To our knowledge this is the first time that they have been found in *Streptomyces*, and they appear to be encoded by genes located in pSCL4 since they were not detected in the *S. clavuligerus* pSCL4^−^ strain.

Cysteine and methionine metabolism in *S. clavuligerus* pSCL4^−^ differs from that of the wild type strain due to the absence of many sulfur metabolism genes that are located either in pSCL4 or at the right end of the chromosome (Additional file 1: Figure S5A, B). However, transcriptomic studies and the formation of metabolites by this strain confirms that enough flow exists to homocysteine and cysteine, the precursors of N-acylhomocysteine lactones and dithiopyrrolones, respectively. The lack of genes *metX* and *metY,* located in at the right end of the chromosome in *S. clavuligerus* pSCL4^−^, suggests that homocysteine formation from homoserine is very low or non-existing in this strain (Additional file 1: Figure S5A, B), but it can be still formed from methionine by the upregulated genes *metK* and *sahH* through S- adenosyl methionine and S-adenosylhomocysteine, respectively. *S. clavuligerus* pSCL4^−^ also lacks the pSCL4-located *metE* gene, to form methionine from homocysteine, but overexpresses the paralogous *metH* gene (Additional file 1: Figure S5A). Cysteine, is formed by the cysteine synthases encoded by SCLAV_4724 and SCLAV_2020, the last one being upregulated at stationary growth phase. Also, the cystathionine-γ-lyase (*cysA*) contributes to cysteine formation (Additional file 1: Figure S5B). However, the genes SCLAV_5668, encoding a cystathionine-γ-synthase, and SCLAV_p1477, encoding a cysteine synthase, do not exist in *S. clavuligerus* pSCL4-; in addition, a gene homologous to *metC,* for a cystathionine β-lyase [35] has not been detected in *S. clavuligerus* genome*.* Sulfur metabolism in *Streptomyces* is still poorly understood, and more biochemical studies are required to know relationship between genes and enzymes and regulatory mechanisms in *Streptomyces* cysteine-methionine metabolism.

Dithiolopyrrolones production in *S. clavuligerus* pSCL4 fits well with the high expression of the holomycin cluster genes [7] of this strain. The metabolomic studies indicated that the largest peak in this group of compounds corresponds to N-propionylholothin. Holomycin was purified from an uncharacterized *S. clavuligerus* mutant by Kenig and Reading [10] and later by De la Fuente et al. [36] and Li and Walsh [37] from mutants lacking the *oppA2* gene; in both cases the purification was made from cultures grown in complex medium. The N-acetyltransferase encoded by *hlmA* has wide substrate specificity, an apparent Km of 15 μM for propionyl-CoA [37] and is able to form N-propionylholothin. The formation of detectable amounts of N-propionylholothin might respond to abundant propanoyl-CoA levels in the cells, in our culture conditions.

In summary, *S. clavuligerus* ATCC 27064 survival is not affected by the lack of 2.1 Mb of genetic information, although the amount and type of secondary metabolites produced and the expression of many genes located in the chromosome is altered. This study provides insight into the cross-talk between the chromosome and the pSCL4 megaplasmid.

## Conclusions

The translocation of DNA fragments from the right arm of the chromosome to plasmids may be frequent in *S. clavuligerus,* as the deletion of the *parAB*_pSCL4_ genes resulted in translocation of a 303 kb DNA fragment. The 1.8 Mb plasmid pSCL4 is dispensable and its lost originates the deletion of the translocated DNA chromosomal fragments, showing in a high plasticity of the *S. clavuligerus* genome. While *S. clavuligerus* strains cured of pSCL4 are viable, this genetic element carries information for secondary metabolites biosynthesis, and for regulatory elements that may modulate the expression of chromosomal genes. Therefore the number and production level of secondary metabolites in the cured strain differs from the wild type *S. clavuligerus*. Of special interest is the lower production of clavulanic acid by *S. clavuligerus* pSCL4^−^ and its high production of sulfur related metabolites, probably due to the alteration of the metabolic pathways leading to sulfur-containing amino acids in this strain.

## Methods

### Strains and culture conditions

*S. clavuligerus* ATCC 27064, as control strain, and the holomycin high producer *S. clavuligerus* pSCL4^−^ strain [6], lacking plasmid pSCL4, were used in transcriptome experiments. *S. clavuligerus parAB::aac*, the parental strain for *S. clavuligerus* pSCL4^−^, was used to locate the translocation of the right arm of the chromosome. The clavulanic acid non-producer, non-sporulating *S. clavuligerus* Δ*claR::aac* [5] was included in sporulation studies. The strains were pregrown in Trypticase Soy Broth (TSB) for 24 h at 28 °C and 220 rpm shaking to an optical density at 600 nm (OD_600_) of 6.5. These seed cultures were used to inoculate (5%, *v*/v) duplicated 500-ml triple-baffled flasks containing 100 ml of defined SA medium [38], and cultures were grown for 72 h under the same conditions. ME medium [39] was used to analyze aerial mycelium formation and sporulation of the strains.

### Antibiotic assays

Clavulanic acid and cephamycin C were quantified as indicated by Pérez-Redondo et al. [40] Holomycin was determined by bioassay against *Micrococcus luteus* ATCC 9341 as described by De la Fuente et al. [36].

### Nucleic acid isolation and RT-qPCR

DNA was isolated as previously described [41]. Relative amount of DNA from the analyzed genes in the right arm of the chromosome, and in plasmids pSCL2 and pSCL4, was quantified by qPCR using 20 ng DNA as described by Lee et al. [42]. The genes analyzed were *parA*_pSCL2_, *parB*_pSCL4,_ SCLAV_5146, 5308, 5482, 5485, 5487, 5491, 5521, 5580, 5585, 5692, 5719, and a region located in the right telomere including the right end of SCLAV_5719 (nucleotides 6,760,214 to 6,760,380), at 12 nt from the end of the chromosome. The chromosomal gene *hrdB* was used as control.

RNA isolation, purification and integrity analysis, and RT-qPCR were performed as indicated previously [5]. The oligonucleotides used in this work are shown in Additional file 1: Table S2.

### Microarray design

The microarrays used in this work have been already described [5]. RNA was extracted from the culture samples at exponential (22.5 h, 46.5 h) and stationary (60 h) phase, and analysis were performed for two biological replicates for each condition (two strains and three growth times). Labeling of RNA preparations with Cy3-dCTP, labeling of genomic DNA as the reference sample with Cy5-dCTP (2.5 pmol/50 μl hybridization solution), and the purification procedures were carried out as described previously [43]. The hybridization conditions, washing, scanning with Agilent Scanner G2565BA, and the quantification of the images were accomplished as previously described [44].

### Transcriptome analysis

Transcriptome analysis was performed as indicated by Martínez-Burgo et al. [5]. The *M*_*g*_ transcription values obtained are proportional to the abundance of the transcript for a particular gene [45] and correspond to the transcription values of the six experimental conditions, mutant versus wild type, corresponding to the three studied growth times. For each gene, *M*_*c*_ values and *P* values were calculated (three sets of values, one for each comparison). *M*_*c*_ values are the binary log of the differential transcription between the mutant and the wild-type strain. The Benjamini-Hochberg (BH) false-discovery rate correction was applied to the *P* values. For each comparison, a result was considered statistically significant if the BH-corrected *P* value was ≤0.05. A positive *M*_*c*_ value indicates upregulation, and a negative *M*_*c*_ value indicates downregulation. In this work, we study those genes with *M*_*c*_ value ≤ −1 in the three sampling times or *M*_*c*_ value ≥1 in the three sampling times.

### Metabolomic analysis

*S. clavuligerus* ATCC 27064 and *S. clavuligerus* pSCL4^−^ were grown in SA medium up to stationary phase as indicated above. For each strain, one 100 ml sample, containing broth and mycelium, was extracted with 1 volume of ethyl acetate with HCl 1%. Extracts from 200 ml of culture were concentrated, and dried samples were resuspended into 100 μL of methanol; 2 μL samples were analyzed in an HPLC Agilent 1200 Rapid Resolution connected to a mass spectrometer maXis from Bruker using a Zorbax SB-C8 (2.1 × 30 mm, 3.5 μm particle size) column. The mobile phase was composed by two solvents containing each ammonium formate 13 mM, and trifluoroacetic acid 0.01%: solvent A, water:acetonitrile 90:10; solvent B, water:acetonitrile 10:90. Elution was performed with a 0.3 ml min^−1^ flow, and the following gradient composition: 90:10 *v*/v 0 min, 0:100 v/v 6 min, 0:100 v/v 8 min, 90:10 v/v 8.1 min, 90:10 v/v 10 min.

Mass spectrometer was adjusted in positive mode ESI (Electrospray Ionization), using 4 kV in the capillary, a drying gas flow of 11 L min^−1^ at 200 °C and a nebulizer pressure of 2.8 bar. Equipment calibration before sample injection was performed using the ions cluster formed by the trifluoroacetic acid (TFA) in the presence of Na^+^ ions. Before the chromatographic front was detectable, every injected sample was recalibrated using TFA-Na. Every chromatographic run was processed using the internal Bruker algorithm for the components extraction, and the more intense peaks, both by positive TIC and for 210 nm absorbance, were analyzed to interpret their exact mass and molecular formula. Both the retention time (RT) and exact mass were used as guideline to search in the High Performance Mass Spectrometry (HPMS) databases from MEDINA Foundation. When a match between the sample RT and mass, and the HPMS databases was found, a search in the Chapman & Hall Dictionary of Natural Products was performed to obtain the formula.

### Bioinformatic analysis

The 294 nt intergenic region between SCLAV_5488 and SCLAV_5489, was analysed by the M. Zuker’s DNA Fold Server (http://unafold.rna.albany.edu/).

## Additional files

Additional file 1: Figure S1. Intergenic region from SCLAV_5488 to SCLAV_5489. Possible hairpin loop formed (ΔG = −85.98 kcal/mol) in the 294 nt excision region. The hairpin loop were predicted according to the M. Zuker’s DNA Fold Server http://unafold. rna.albany.edu/). **Figure S2.** Antibiotics production. *S. clavuligerus* ATCC27064 (white circles) and *S. clavuligerus* pSCL4^−^ (black circles) were grown in SA medium, and production of clavulanic acid (left panel), cephamycin C (central panel) and holomycin (right panel) was quantified. **Figure S3.** RT-qPCR validation of the microarray data. (A) Genes tested: comparison of the data obtained for each gene analyzed in microarrays experiment (Mc values) and by RT-qPCR [log_2_ -2E(ΔΔCt)]. (B) Representation of the correlation between the results showed in panel A. **Figure S4.** MS and UV absortion spectra of the compounds detected in *S. clavuligerus* and the pSCL4^−^ mutant. Only holomycin and N-propionyl holothin, detected in the mutant, have been described previously. **Figure S5.** Transcriptomic data of Methionine and Cysteine Metabolism Genes**.** A) Change of expression level of genes related to methionine or cysteine metabolism in *S. clavuligerus* pSCL4-. Genes not present in *S. clavuligerus* pSCL4^−^ since they are in the megaplasmid are indicated with double asterisk (**); those genes present among the 231 CDS absent at the right arm of the chromosome are indicated with single asterisk (*). B) Pathways of methionine and cysteine metabolism in *Streptomyces*. Open arrows indicated upregulated genes in the pSCL4- strain. Steps carried by enzymes encoded by genes not present are marked with a black circle. **Table S1.** Effect of lack of pSCL4^−^ in genes involved in cell envelope formation and morphological differentiation. **Table S2.** Oligonucleotides used in this work. (PDF 2056 kb)

## Abbreviations

CDS: Coding sequences
SMC: Secondary metabolism gene cluster

## References

[1] Medema MH, Trefzer A, Kovalchuk A, van den Berg M, Müller U, Heijne W, Wu L, Alam MT, Ronning CM, Nierman WC, Bovenberg RA, Breitling R, Takano E (2010). The sequence of a 1.8-Mb bacterial linear plasmid reveals a rich evolutionary reservoir of secondary metabolic pathways. Genome Biol Evol.

[2] Netolitzky DJ, Wu X, Jensen SE, Roy KL (1995). Giant linear plasmids of beta-lactam antibiotic producing *Streptomyces*. FEMS Microbiol Lett.

[3] Charusanti P, Fong NL, Nagarajan H, Pereira AR, Li HJ, Abate EA, Su Y, Gerwick WH, Palsson BO (2012). Exploiting adaptive laboratory evolution of *Streptomyces clavuligerus* for antibiotic discovery and overproduction. PLoS One.

[4] Lorenzana LM, Pérez-Redondo R, Santamarta I, Martín JF, Liras P (2004). Two oligopeptide-permease-encoding genes in the clavulanic acid cluster of *Streptomyces clavuligerus* are essential for production of the β-lactamase inhibitor. J Bacteriol.

[5] Martínez-Burgo Y, Álvarez-Álvarez R, Rodríguez-García A, Liras P (2015). The Pathway-Specific Regulator ClaR of *Streptomyces clavuligerus* Has a Global Effect on the Expression of Genes for Secondary Metabolism and Differentiation. Appl Environ Microbiol.

[6] Álvarez-Álvarez R, Rodríguez-García A, Martínez-Burgo Y, Robles-Reglero V, Santamarta I, Pérez-Redondo R, Martín JF, Liras P (2014). A 1.8-Mb-reduced *Streptomyces clavuligerus* genome, relevance for secondary metabolism and differentiation. Appl Microbiol Biotechnol.

[7] Robles-Reglero V, Santamarta I, Álvarez-Álvarez R, Martín JF, Liras P (2013). Transcriptional analysis and proteomics of the holomycin gene cluster in overproducer mutants of *Streptomyces clavuligerus*. J Biotechnol.

[8] Blin K, Kazempour D, Wohlleben W, Weber T (2014). Improved Lanthipeptide detection and prediction for antiSMASH. PLoS One.

[9] Weber T, Blin K, Duddela S, Krug D, Kim HU, Bruccoleri R, Lee SY, Fischbach MA, Müller R, Wohlleben W, Breitling R, Takano E (2015). Medema, MH. antiSMASH 3.0-a comprehensive resource for the genome mining of biosynthetic gene clusters. Nucleic Acids Res.

[10] Kenig M, Reading C (1979). Holomycin and an antibiotic (MM 19290) related to tunicamycin, metabolites of *Streptomyces clavuligerus*. J Antibiot.

[11] Barona-Gómez F, Wong U, Giannakopulos AE, Derrick PJ, Challis GL (2004). Identification of a cluster of genes that directs desferrioxamine biosynthesis in *Streptomyces coelicolor* M145. J Am Chem Soc.

[12] Paget MSB, Leibovitz E, Buttner MJ (1999). A putative two-component signal transduction system regulates sigmaE, a sigma factor required for normal cell wall integrity in *Streptomyces coelicolor* A3(2). Mol Microbiol.

[13] Hahn JS, Oh SY, Roe JH (2002). Role of OxyR as a peroxide-sensing positive regulator in *Streptomyces coelicolor* A3(2). J Bacteriol.

[14] Kim MS, Dufour YS, Yoo JS, Cho YB, Park JH, Nam GB, Kim HM, Lee KL, Donohue TJ, Roe JH (2012). Conservation of thiol-oxidative stress responses regulated by SigR orthologues in actinomycetes. Mol Microbiol.

[15] Cho YH, Lee EJ, Ahn BE, Roe JH (2001). SigB, an RNA polymerase sigma factor required for osmoprotection and proper differentiation of *Streptomyces coelicolor*. Mol Microbiol.

[16] Shu D, Chen L, Wang W, Yu Z, Ren C, Zhang W, Yang S, Lu Y, Jiang W (2009). AfsQ1-Q2-sigQ is a pleiotropic but conditionally required signal transduction system for both secondary metabolism and morphological development in *Streptomyces coelicolor*. Appl Microbiol Biotechnol.

[17] Uguru GC, Stephens KE, Stead JA, Towle JE, Baumberg S, McDowall KJ (2005). Transcriptional activation of the pathway-specific regulator of the actinorhodin biosynthetic genes in *Streptomyces coelicolor*. Mol Microbiol.

[18] Chen L, Lu Y, Chen J, Zhang W, Shu D, Qin Z, Yang S, Jiang W (2008). Characterization of a negative regulator AveI for avermectin biosynthesis in *Streptomyces avermitilis* NRRL8165. Appl Microbiol Biotechnol.

[19] Kang SH, Huang J, Lee HN, Hur YA, Cohen SN, Kim ES (2007). Interspecies DNA microarray analysis identifies WblA as a pleiotropic down-regulator of antibiotic biosynthesis in *Streptomyces*. J Bacteriol.

[20] Gál J, Szvetnik A, Schnell R, Kálmán M (2002). The *metD* D-methionine transporter locus of *Escherichia coli* is an ABC transporter gene cluster. J Bacteriol.

[21] Noens EE, Mersinias V, Traag BA, Smith CP, Koerten HK, van Wezel GP (2005). (2005). SsgA-like proteins determine the fate of peptidoglycan during sporulation of *Streptomyces coelicolor*. Mol Microbiol.

[22] Pivetti CD, Yen MR, Miller S, Busch W, Tseng YH, Booth IR, Saier MHJr. Two families of mechanosensitive channel proteins. Microbiol Mol Biol Rev 2003; 67: 66-85. 10.1128/MMBR.67.1.66-85.2003 PMC150521 12626684

[23] Clark LC, Seipke RF, Prieto P, Willemse J, van Wezel GP, Hutchings MI, Hoskisson PA (2013). Mammalian cell entry genes in *Streptomyces* may provide clues to the evolution of bacterial virulence. Sci Rep.

[24] Kodani S, Hudson ME, Durrant MC, Buttner MJ, Nodwell JR, Willey JM (2004). The SapB morphogen is a lantibiotic-like peptide derived from the product of the developmental gene *ramS* in *Streptomyces coelicolor*. Proc Natl Acad Sci U S A.

[25] Lee HS, Shin HJ, Jang KH, Kim TS, Oh KB, Shin J (2005). Cyclic peptides of the nocardamine class from a marine-derived bacterium of the genus *Streptomyces*. J Nat Prod.

[26] Okamura K, Soga K, Shimauchi Y, Ishikura T, Lein J (1977). Holomycin and N-propionylholothin, antibiotics produced by a cephamycin C producer. J Antibiot.

[27] Song JY, Jeong H, Yu DS, Fischbach MA, Park HS, Kim JJ, Seo JS, Jensen SE, Oh TK, Lee KJ, Kim JF (2010). *Draft genome sequence of Streptomyces clavuligerus* NRRL 3585, a producer of diverse secondary metabolites. J Bacteriol.

[28] Fischer G, Wenner T, Decaris B, Leblond P (1998). Chromosomal arm replacement generates a high level of intraspecific polymorphism in the terminal inverted repeats of the linear chromosomal DNA of *Streptomyces ambofaciens*. Proc Natl Acad Sci U S A.

[29] Musialowski MS, Flett F, Scott GB, Hobbs G, Smith CP, Oliver SG (1994). Functional evidence that the principal DNA replication origin of the *Streptomyces coelicolor* chromosome is close to the *dnaA*-*gyrB* region. J Bacteriol.

[30] Volff JN, Viell P, Altenbucher J (1997). Artifitial circularization of the chromosome with concomitant deletion of its terminal inverted repeats enhances genetic instability and genome rearrangement in *Streptomyces lividans*. Mol Gen Genet.

[31] Volff JN, Viell P, Altenbucher J (2000). A new beginning with new ends, linearisation of circular chromosomes during bacterial evolution. FEMS Microbiol Lett.

[32] Kim HS, Lee YJ, Lee CK, Choi SU, Yeo SH, Hwang YI, Yu TS, Kinoshita H, Nihira T (2004). Cloning and characterization of a gene encoding the gamma-butyrolactone autoregulator receptor from *Streptomyces clavuligerus*. Arch Microbiol.

[33] Santamarta I, Pérez-Redondo R, Lorenzana LM, Martín JF, Liras P (2005). Different proteins bind to the butyrolactone-receptor protein ARE sequence located upstream of the regulatory *ccaR* gene of *S. clavuligerus*. Mol Microbiol.

[34] Davidova OK, Deriabin DG, Nikiian AN, El’-Registan GI (2005). Mechanisms of interaction between DNA and chemical analogues of microbial anabiosis autoinducers. Mikrobiologiia.

[35] Kulkarni A, Zeng Y, Zhou W, VanLanen S, Zhang W, Chen S (2016). A branch point of *Streptomyces* sulfur amino acid metabolism controls the production of albomycin. Appl Environ Microbiol.

[36] De la Fuente A, Lorenzana LM, Martín JF, Liras P (2002). Mutants of *Streptomyces clavuligerus* with disruptions in different genes for clavulanic acid biosynthesis produce large amounts of holomycin, possible crossregulation of two unrelated secondary metabolic pathways. J Bacteriol.

[37] Li B, Walsh CT (2010). Identification of the gene cluster for the dithiolopyrrolone antibiotic holomycin in *Streptomyces clavuligerus*. Proc Natl Acad Sci U S A.

[38] Aidoo KA, Wong A, Alexander DC, Rittammer RA, Jensen SE (1994). Cloning, sequencing and disruption of a gene from *Streptomyces clavuligerus* involved in clavulanic acid biosynthesis. Gene.

[39] Sánchez L, Braña A (1996). Cell density influences antibiotic biosynthesis in *Streptomyces clavuligerus*. Microbiology.

[40] Pérez-Redondo R, Rodríguez-García A, Martín JF, Liras P (1998). The *claR* gene of *Streptomyces clavuligerus*, encoding a LysR-type regulatory protein controlling clavulanic acid biosynthesis, is linked to the clavulanate-9-aldehyde reductase (*car*) gene. Gene.

[41] Pospiech A, Neumann B (1995). A versatile quick-prep of genomic DNA from gram-positive bacteria. Trends Genet.

[42] Lee C, Kim J, Shin SG, Hwang S (2006). Absolute and relative qPCR quantification of plasmid copy number in *Escherichia coli*. J Biotechnol.

[43] Álvarez-Álvarez R, Rodríguez-García A, Santamarta I, Pérez-Redondo R, Prieto-Domínguez A, Martínez-Burgo Y, Liras P (2014). Transcriptomic analysis of *Streptomyces clavuligeru*s Δ*ccaR, tsr,* effects of the cephamycin C-clavulanic acid cluster regulator CcaR on global regulation. Microb Biotechnol.

[44] Yagüe P, Rodríguez-García A, López-García MT, Rioseras B, Martín JF, Sánchez J, Manteca A (2014). Transcriptomic analysis of liquid nonsporulating *Streptomyces coelicolor* cultures demonstrates the existence of a complex differentiation comparable to that occurring in solid sporulating cultures. PLoS One.

[45] Mehra S, Lian W, Jayapal KP, Charaniya SP, Sherman DH, Hu W-S (2006). A framework to analyze multiple time series data, a case study with *Streptomyces coelicolor*. J Ind Microbiol Biotechnol.

