# Supplementary material for: Discovering the potential of S. clavuligerus for bioactive compound production: cross-talk between the chromosome and the pSCL4 megaplasmid
Source: BMC Genomics. 2017 Nov 25;18:907. doi: 10.1186/s12864-017-4289-y (PMC5702194; doi:10.1186/s12864-017-4289-y)
Supplement: Supplementary file 1 — Intergenic region from SCLAV_5488 to SCLAV_5489. Possible hairpin loop formed (ΔG = −85.98 kcal/mol) in the 294 nt excision region. The hairpin loop were predicted according to the M. Zuker’s DNA Fold Server http://unafold. rna.albany.edu/). Figure S2. Antibiotics production. S. clavuligerus ATCC27064 (white circles) and S. clavuligerus pSCL4− (black circles) were grown in SA medium, and production of clavulanic acid (left panel), cephamycin C (central panel) and holomycin (right panel) was quantified. Figure S3. RT-qPCR validation of the microarray data. (A) Genes tested: comparison of the data obtained for each gene analyzed in microarrays experiment (Mc values) and by RT-qPCR [log2 -2E(ΔΔCt)]. (B) Representation of the correlation between the results showed in panel A. Figure S4. MS and UV absortion spectra of the compounds detected in S. clavuligerus and the pSCL4− mutant. Only holomycin and N-propionyl holothin, detected in the mutant, have been described previously. Figure S5. Transcriptomic data of Methionine and Cysteine Metabolism Genes. A) Change of expression level of genes related to methionine or cysteine metabolism in S. clavuligerus pSCL4-. Genes not present in S. clavuligerus pSCL4− since they are in the megaplasmid are indicated with double asterisk (**); those genes present among the 231 CDS absent at the right arm of the chromosome are indicated with single asterisk (*). B) Pathways of methionine and cysteine metabolism in Streptomyces. Open arrows indicated upregulated genes in the pSCL4- strain. Steps carried by enzymes encoded by genes not present are marked with a black circle. Table S1. Effect of lack of pSCL4− in genes involved in cell envelope formation and morphological differentiation. Table S2. Oligonucleotides used in this work. (PDF 2056 kb) [file 12864_2017_4289_MOESM1_ESM.pdf]

Additional file 1

Discovering the potential of *Streptomyces clavuligerus* for bioactive compound production: cross-talk between the chromosome and the pSCL4 megaplasmid

Rubén Álvarez-Álvarez*[1], Yolanda Martínez-Burgo*[1], Antonio Rodríguez-García[1,2], Paloma Liras[1]

1) Microbiology Section, Faculty of Biological and Environmental Sciences, University of León, León, Spain. 2) Institute of Biotechnology of León, INBIOTEC, León, Spain.

***Authors with equal contributions.**

Corresponding author: Paloma Liras paloma.liras@unileon.es.

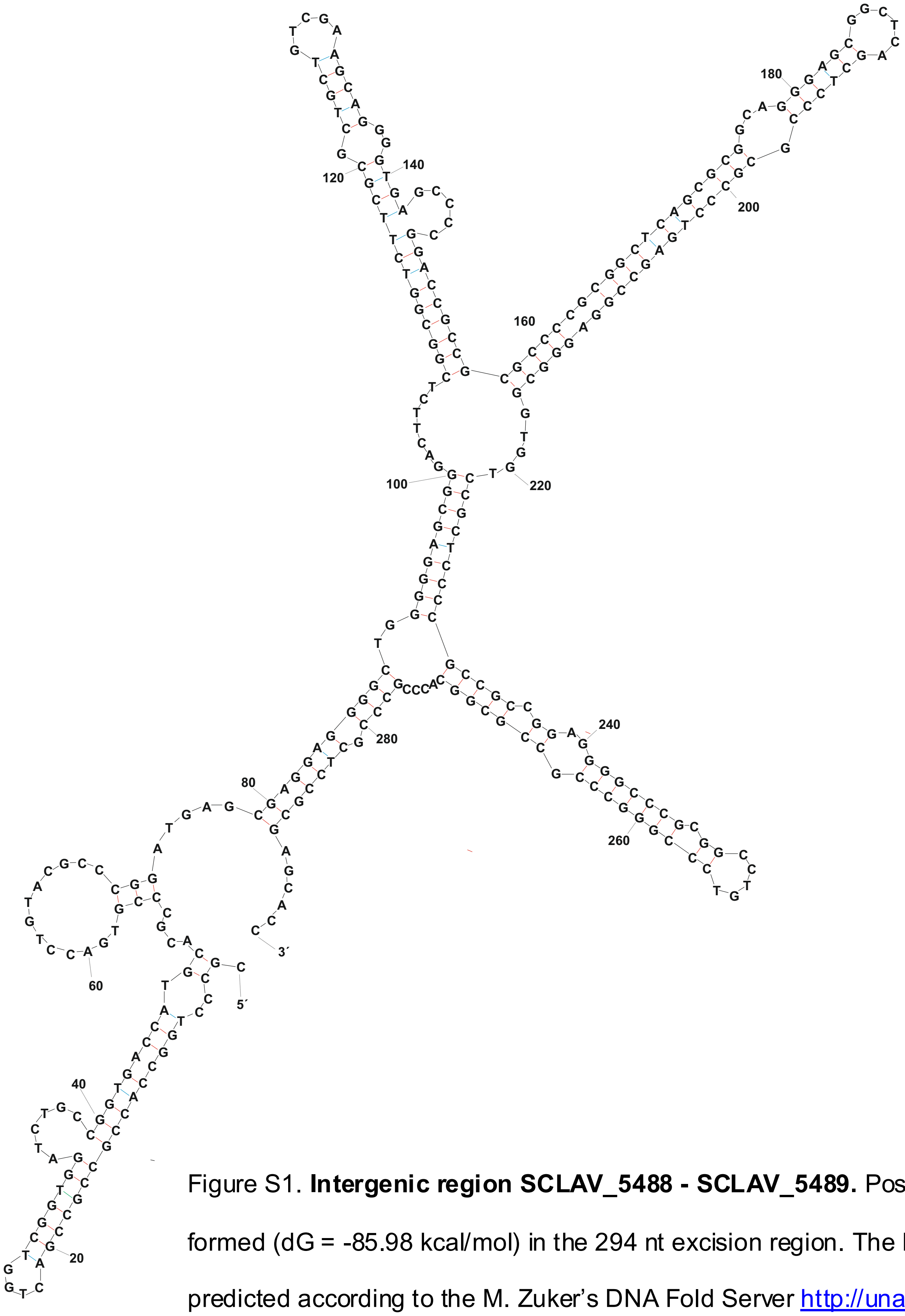

Figure S1. **Intergenic region SCLAV_5488 - SCLAV_5489.** Possible hairpin lo[illegible] formed (dG = -85.98 kcal/mol) in the 294 nt excision region. The hairpin loop wa[illegible] predicted according to the M. Zuker's DNA Fold Server http://unafold.rna.albany[illegible] edu/).

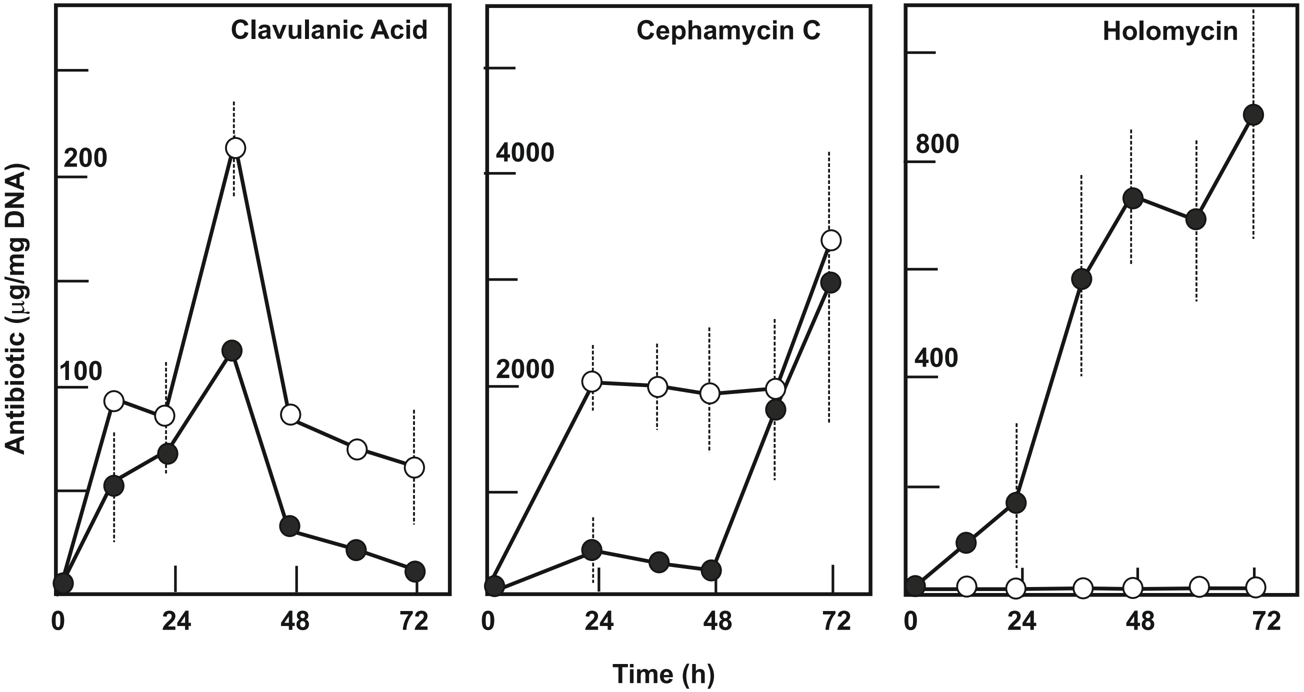

Figure S2. **Antibiotics production**. *S. clavuligerus* ATCC27064 (white circles) and *S. clavuligerus* pSCL4$^-$ (black circles) were grow in SA medium, and production of clavulanic acid (left panel), cephamycin C (central panel) and holomycin (right panel) was quantified.

Fig. S2 Alvarez-Alvarez et al

A

Gene	Product	M_c	$\log_2 -2^{\Delta\Delta C_t}$
SCLAV_0014	Modular polyketide synthase	5.58	7.48
SCLAV_0785	Methionine import ATP-binding protein (MetN)	5.01	6.54
SCLAV_3410	TetR-family transcriptional regulator	-2.36	-1.23
SCLAV_4035	ABC transporter solute binding protein	3.83	5.32
SCLAV_4183	OppA2: Oligopeptide permease	-1.67	-1.07
SCLAV_4199	PcbC: cehamycin C biosynthesis	-1.84	-0.99
SCLAV_4464	LuxR-family transcriptional regulator	-2.35	-2.59
SCLAV_4484	Methylmalonyl-CoA decarboxylase α unit	2.69	2.86
SCLAV_4650	AraC-family transcriptional regulator	2.73	4.73
SCLAV_4764	Oxidoreductase	-2.96	-2.59
SCLAV_5267	HlmA: holomycin biosynthesis	4.01	5.91
SCLAV_5308	Transcription factor	-2.89	-2.00

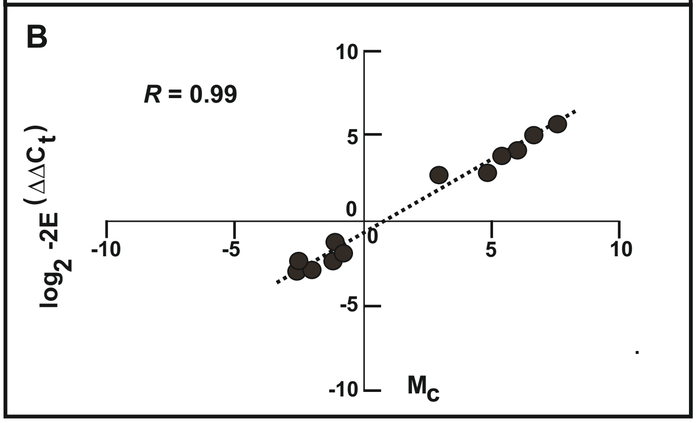

Figure S3. **RT-qPCR validation of the microarray data.** (A) Genes tested: comparison of the data obtained for each gene analyzed in microarrays experiment (M_c values) and by RT-qPCR [$\log_2$ -2E($\Delta\Delta$Ct)]. (B) Representation of the correlation between the results showed in panel A.

Fig S3. Alvarez-Alvarez et al

Fig. S4

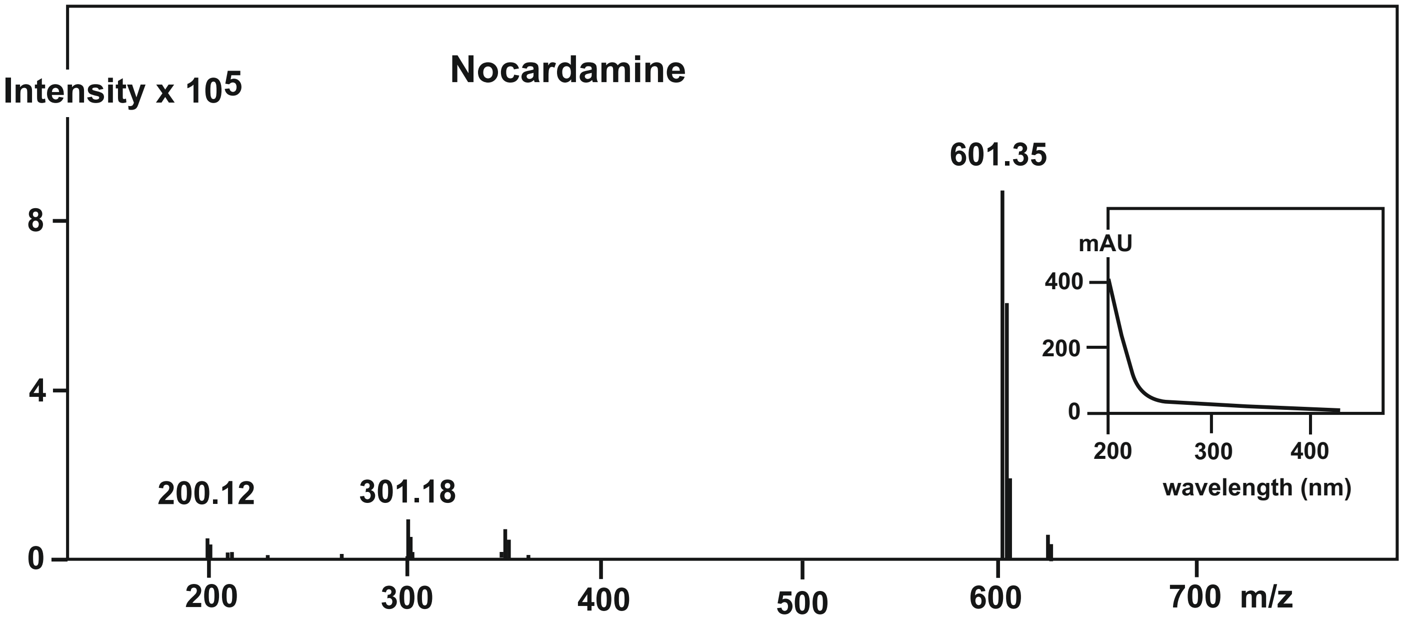
Nocardamine
Intensity x 10^5
0
4
8
200.12
301.18
601.35
200
300
400
500
600
700 m/z
mAU
400
200
0
200
300
400
wavelength (nm)

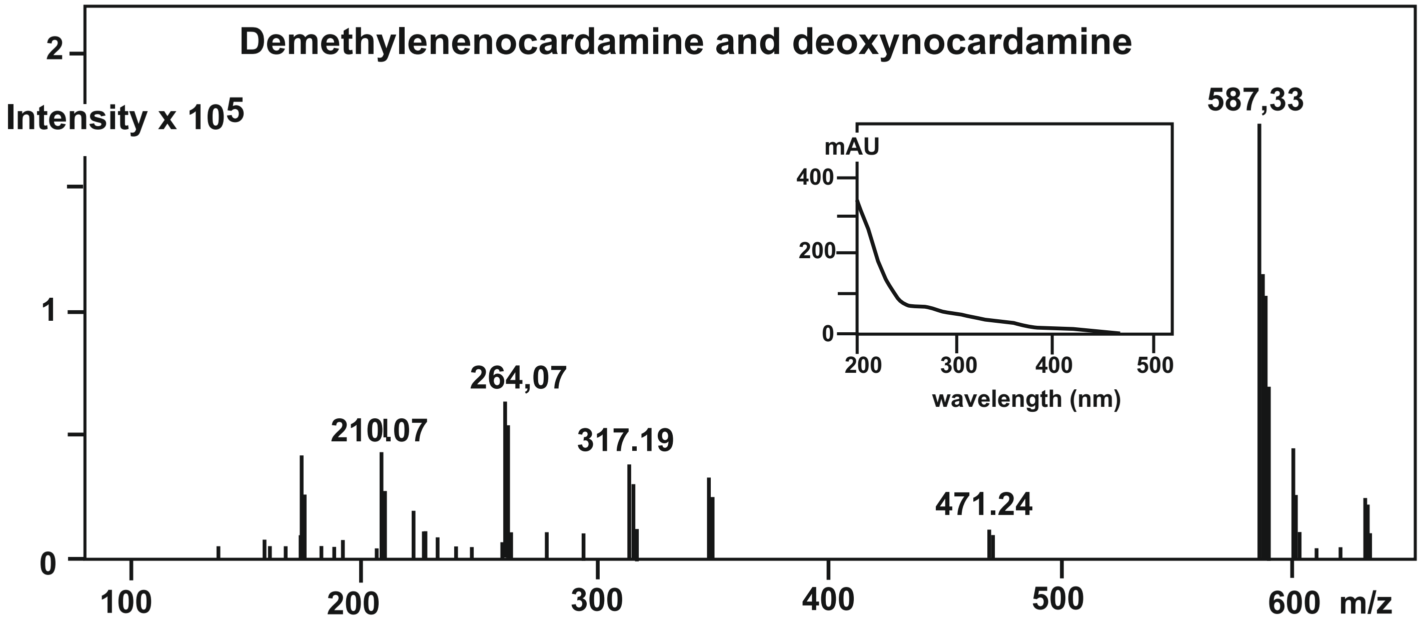
Demethylenenocardamine and deoxynocardamine
Intensity x 10^5
0
1
2
210.07
264,07
317.19
471.24
587,33
100
200
300
400
500
600 m/z
mAU
400
200
0
200
300
400
500
wavelength (nm)

Fig. S4

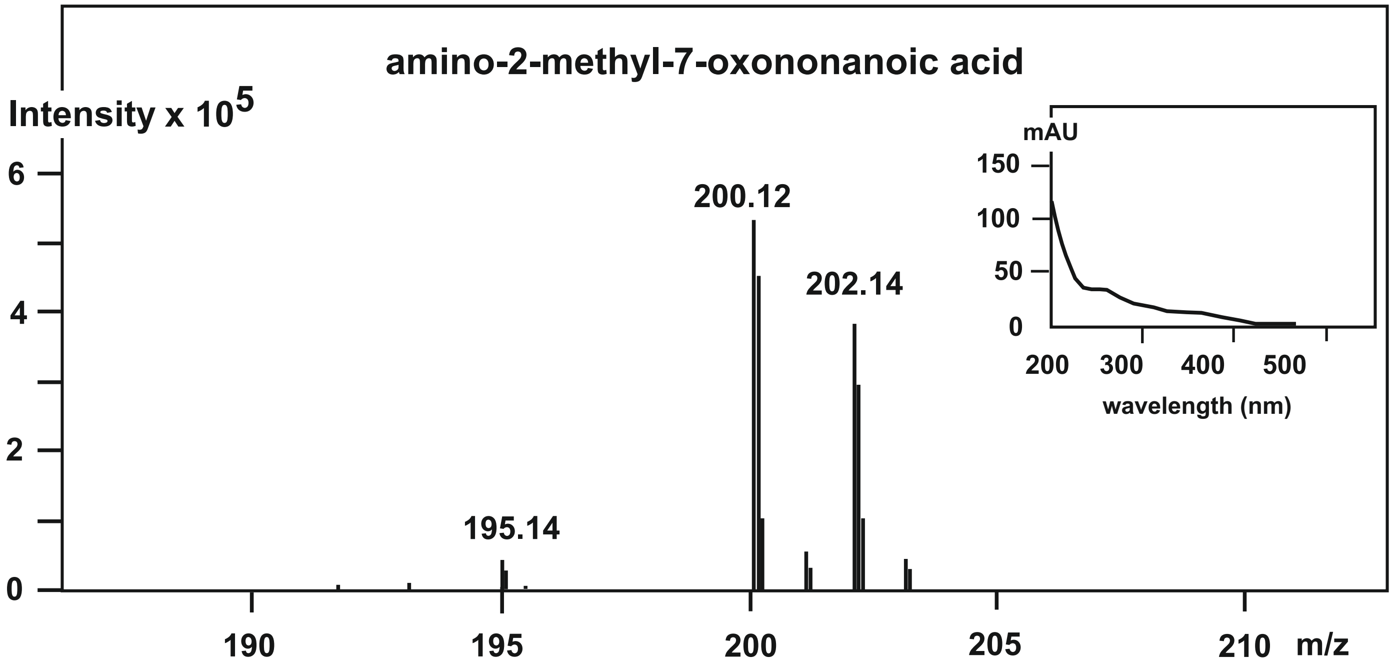

amino-2-methyl-7-oxononanoic acid
Intensity x 10^5
6
4
2
0
200.12
202.14
195.14
190
195
200
205
210
m/z
mAU
150
100
50
0
200
300
400
500
wavelength (nm)

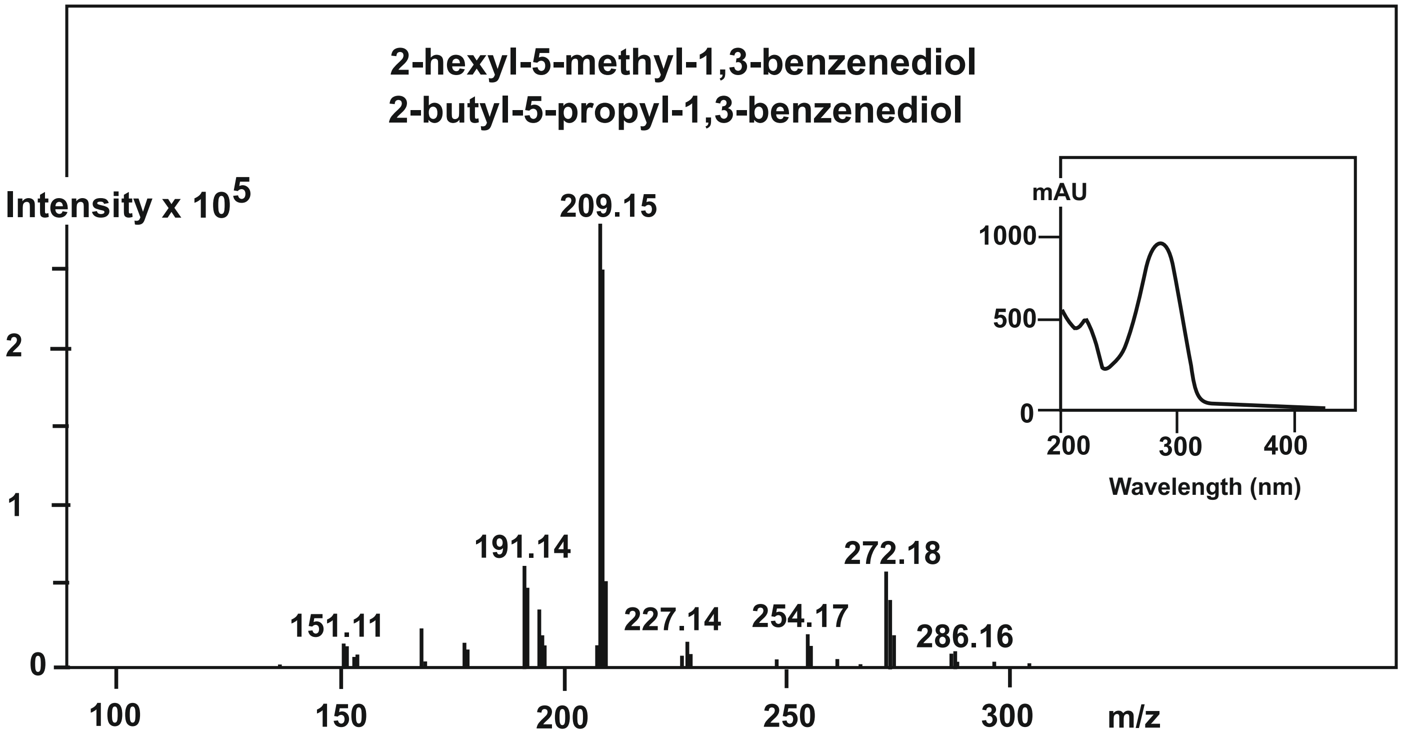

2-hexyl-5-methyl-1,3-benzenediol
2-butyl-5-propyl-1,3-benzenediol
Intensity x 10^5
2
1
0
209.15
191.14
151.11
227.14
254.17
272.18
286.16
100
150
200
250
300
m/z
mAU
1000
500
0
200
300
400
Wavelength (nm)

Fig. S4

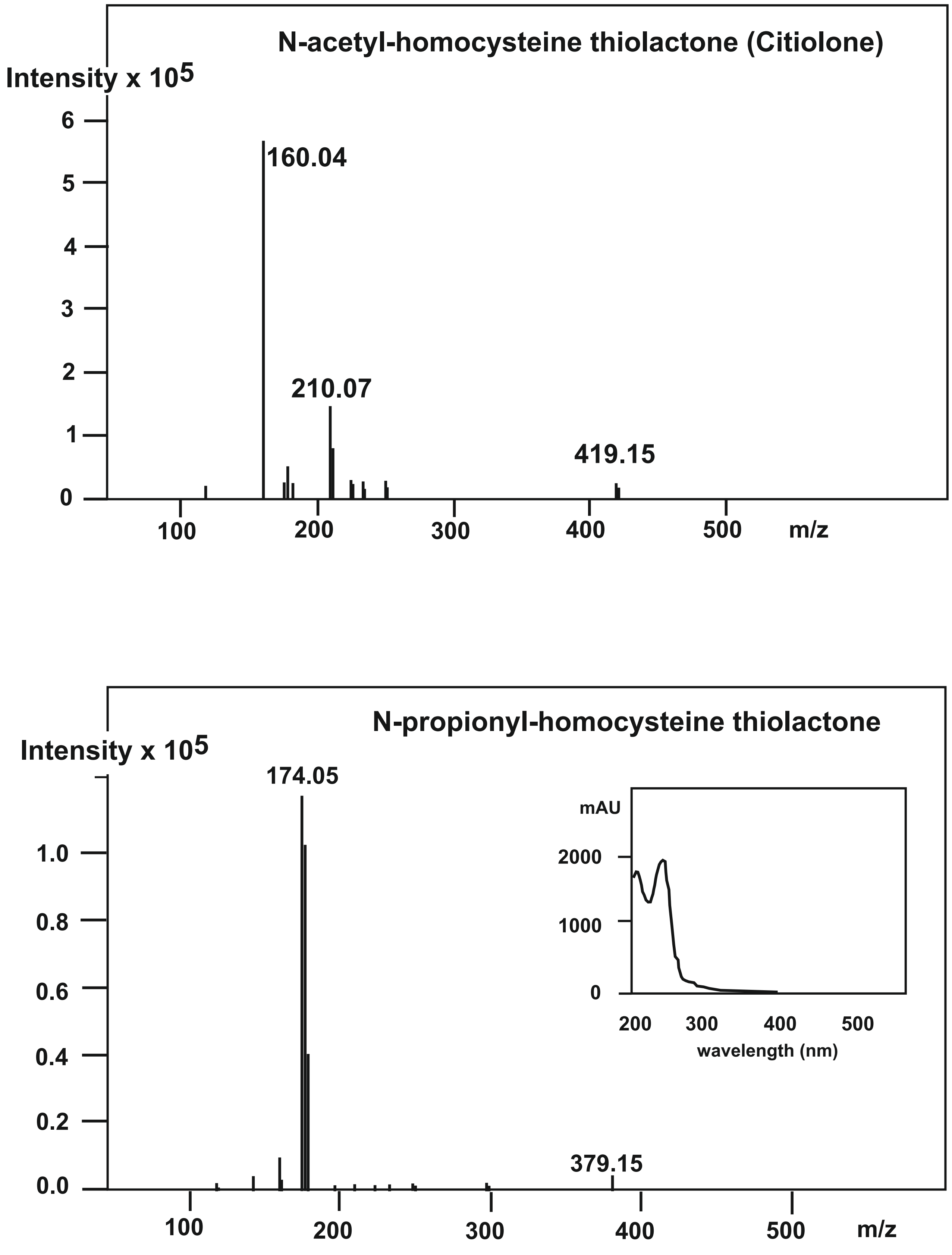
N-acetyl-homocysteine thiolactone (Citiolone)
Intensity x 10^5
6
5
4
3
2
1
0
160.04
210.07
419.15
100
200
300
400
500
m/z
N-propionyl-homocysteine thiolactone
Intensity x 10^5
174.05
1.0
0.8
0.6
0.4
0.2
0.0
379.15
100
200
300
400
500
m/z
mAU
2000
1000
0
200
300
400
500
wavelength (nm)

Fig. S4

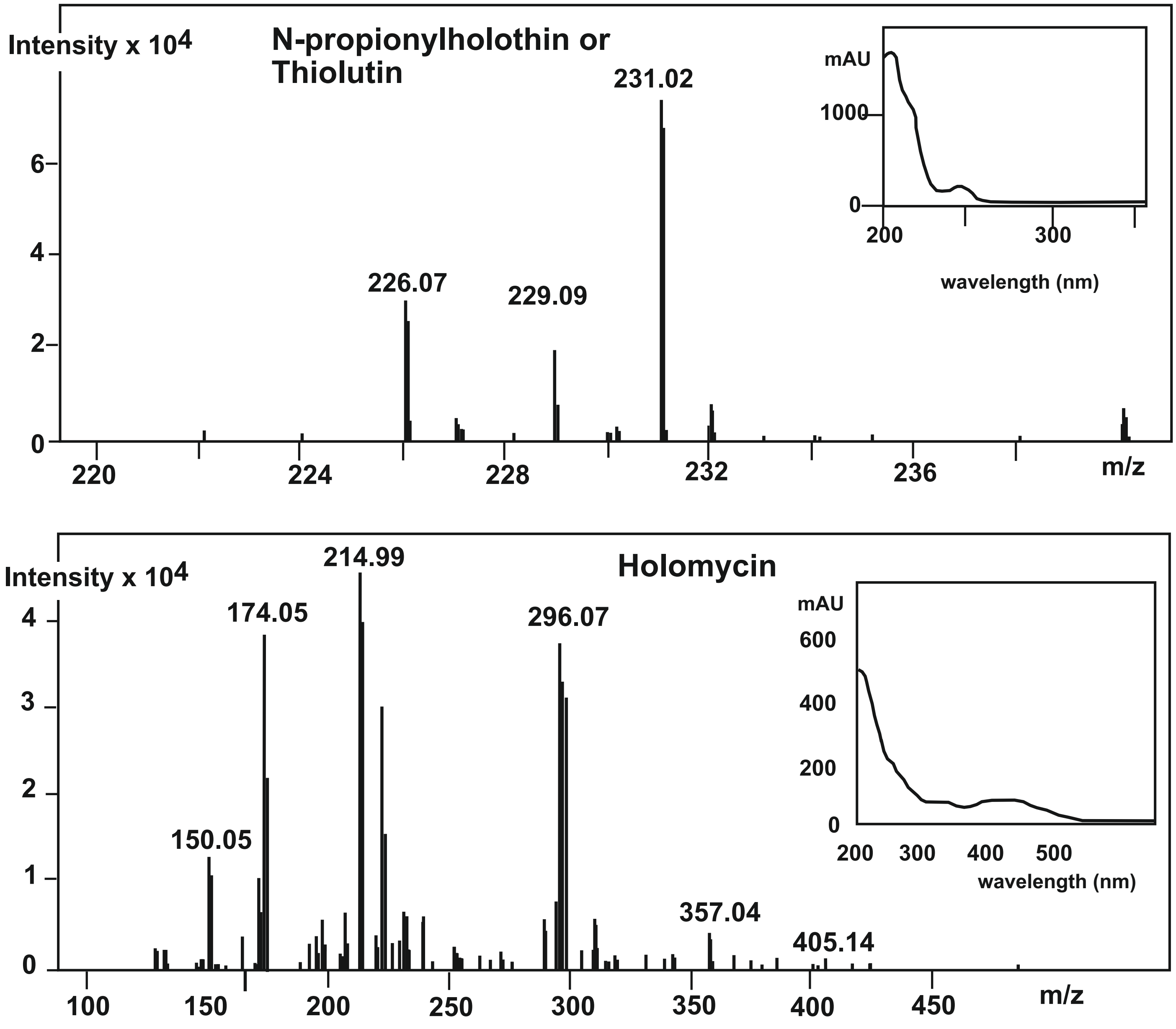

Fig. S4. **MS and UV absorption spectra of the new compounds detected in *S. clavuligerus* ATCC 27064 and the pSCL4^{-} mutant (see Fig. 6).** Only holomycin and N-propionylholothin have been described previously to be produced by *S. clavuligerus* strains.

Figure S5. Methionine and Cysteine Metabolism in *S. clavuligerus* pSCL4^{-}

A. Transcriptomic of Methionine and Cysteine metabolism genes

Gene	SCLAV	Product	Mc			BH			Change of expression (fold)		
			22.5 h	46.5 h	60 h	22.5 h	46.5 h	60 h	22.5 h	46.5 h	60 h
metK	SCLAV_0705	S-adenosylmethionine synthetase	0.75	0.81	0.91	$3.48E^{-02}$	$1.71E^{-02}$	$6.33E^{-03}$	1.68	1.75	1.88
metH	SCLAV_0875	Methionine synthase	3.04	3.60	4.73	$3.35E^{-04}$	$3.62E^{-05}$	$8.99E^{-07}$	8.24	12.14	26.59
sahH	SCLAV_2107	Adenosylhomocysteinase	2.67	2.79	3.16	$1.33E^{-04}$	$5.68E^{-05}$	$9.45E^{-06}$	6.37	6.93	8.93
	SCLAV_2199	Cystathionine beta-synthase	-0.52	-0.40	0.66	$1.50E^{-01}$	$2.53E^{-01}$	$4.16E^{-02}$	0.70	0.76	1.58
cysA	SCLAV_2869	Cystathionine gamma-lyase	0.13	0.10	0.34	$7.73E^{-01}$	$8.01E^{-01}$	$3.01E^{-01}$	1.10	1.07	1.26
metB	SCLAV_3851	Cystathionine gamma-synthase	-0.33	0.07	0.91	$3.64E^{-01}$	$8.33E^{-01}$	$1.42E^{-03}$	0.79	1.05	1.88
metX	SCLAV_5558**	Homoserine O-acetyltransferase	-6.40	-6.73	-3.78	$3.71E^{-05}$	$1.40E^{-05}$	$3.94E^{-03}$	0.01	0.01	0.07
	SCLAV_5559**	O-acetylhomoserine aminocarboxypropyltransferase	-8.98	-9.53	-5.89	$1.80E^{-05}$	$5.69E^{-06}$	$7.35E^{-04}$	0.00	0.00	0.02
metE	SCLAV_p0930*	Homocysteine methyltransferase B12-dependet	Absent								
cysK	SCLAV_0274	Cysteine synthase/cystathionine beta-synthase	1.76	1.88	1.23	$7.00E^{-04}$	$2.60E^{-04}$	$8.77E^{-03}$	3.40	3.68	2.35
	SCLAV_2020	Cysteine synthase	-0.74	-0.15	1.27	$1.17E^{-02}$	$6.50E^{-01}$	$3.59E^{-05}$	0.60	0.90	2.42
	SCLAV_4724	Putative cysteine synthase	1.71	3.89	4.30	$1.75E^{-02}$	$4.95E^{-06}$	$6.77E^{-07}$	3.26	14.79	19.67
	SCLAV_5668**	Cystathionine gamma-synthase	-3.17	-3.14	-3.78	$2.31E^{-04}$	$1.86E^{-04}$	$1.55E^{-05}$	0.11	0.11	0.07
	SCLAV_5714**	Cysteine desulfurase	-3.39	-3.50	-3.12	$1.16E^{-05}$	$4.11E^{-06}$	$6.90E^{-06}$	0.10	0.09	0.11
	SCLAV_p0023*	Cysteine desulfurase, SufS subfamily	Absent								
	SCLAV_p1477*	Cysteine synthase	Absent								

Fg S5 B

S5-B. Methionine and cysteine metabolism in *S. clavuligerus*

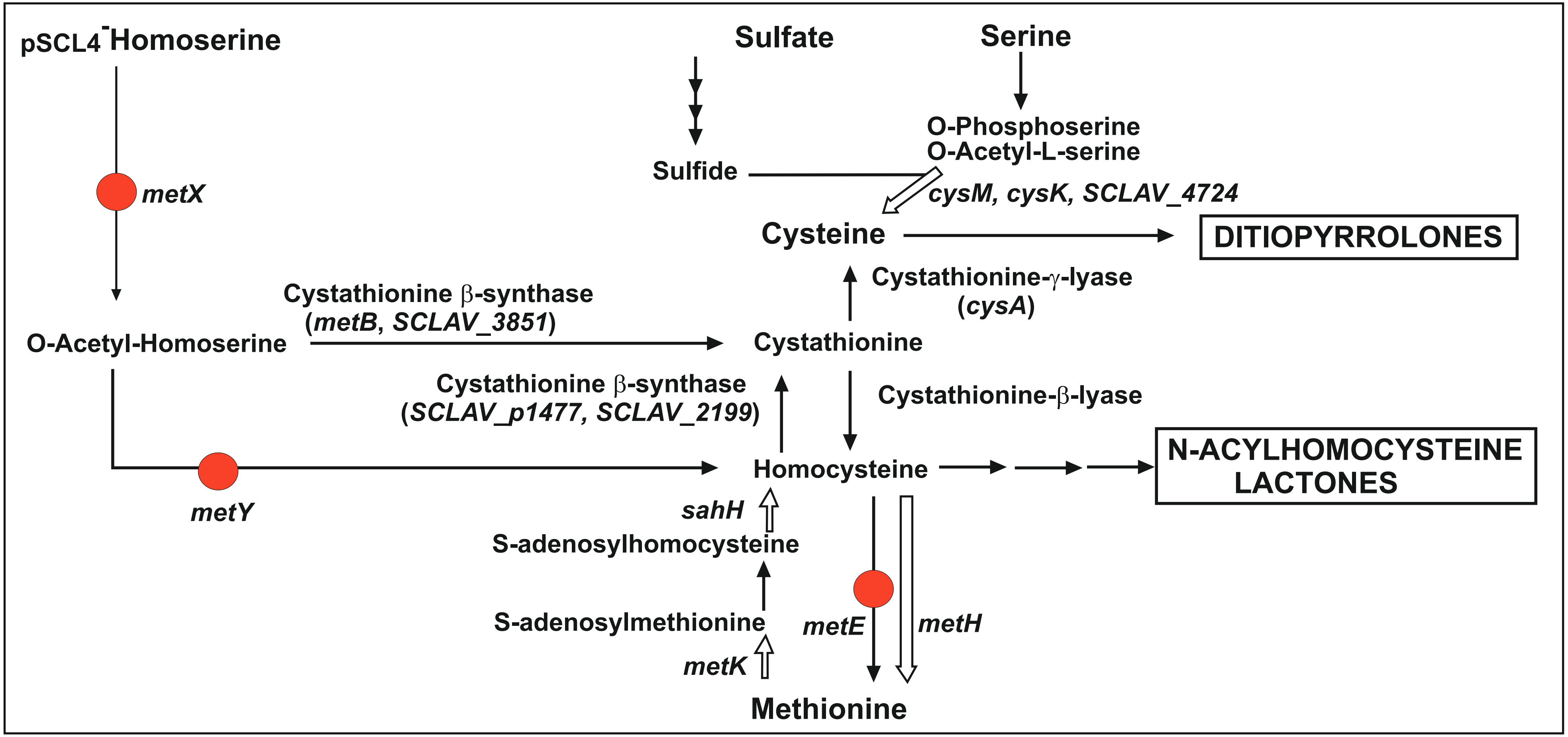

Figure S5. **Transcriptomic data of Methionine and Cysteine Metabolism Genes.** A) Change of expression level of genes related to methionine or cysteine metabolism in *S. clavuligerus* pSCL4-. Genes not present in *S. clavuligerus* pSCL4- since they are in the megaplasmid are indicated with double asterisk (**); those genes present among the 231 CDS absent at the right arm of the chromosome are indicated with single asterisk (*). B) Pathways of methionine and cysteine metabolism in *Streptomyces*. Open arrows indicated upregulated genes in the pSCL4-minus strain. Steps carried by enzymes encoded by genes not present are marked with a black circle.

Table S1. Effect of lack of pSCL4- in genes involved in cell envelope formation and morphological differentiation.

SCLAV	Product	M_c			BH-corrected *P* value			Fold change		
		**22.5 h**	**46.5 h**	**60 h**	**22.5 h**	**46.5 h**	**60 h**	**22.5 h**	**46.5 h**	**60 h**
**Overexpressed**										
SCLAV_0315	UDP-N-acetylglucosamine 2-epimerase	2.67	2.31	1.54	$2.31E^{-05}$	$6.90E^{-05}$	$3.07E^{-03}$	6.35	4.97	2.91
SCLAV_0316	Putative N-acetyl-glucosamine transferase	2.04	2.11	2.51	$5.47E^{-04}$	$2.73E^{-04}$	$2.68E^{-05}$	4.11	4.33	5.71
SCLAV_0874	Transferase	1.03	1.10	2.26	$1.43E^{-02}$	$6.49E^{-03}$	$2.50E^{-06}$	2.04	2.15	4.80
SCLAV_1824	MscS mechanosensitive ion channel	1.48	1.77	2.49	$5.45E^{-04}$	$5.57E^{-05}$	$6.12E^{-07}$	2.80	3.41	5.60
SCLAV_3942	Putative penicillin-binding protein	1.87	1.84	2.15	$6.71E^{-04}$	$5.79E^{-04}$	$8.07E^{-05}$	3.67	3.59	4.42
SCLAV_4710	Peptidase	1.69	2.43	3.52	$2.02E^{-03}$	$3.11E^{-05}$	$1.84E^{-07}$	3.22	5.38	11.50
SCLAV_5204	Putative SsgD protein	2.35	2.73	2.60	$6.73E^{-06}$	$1.22E^{-06}$	$2.42E^{-07}$	5.10	6.66	6.04
**Downexpressed**										
SCLAV_0202	Putative non-hemolytic phospholipase C	-1.33	-1.82	-1.80	$1.80E^{-04}$	$4.70E^{-06}$	$2.43E^{-06}$	0.40	0.28	0.29
SCLAV_2383	Putative penicillin-binding exported protein	-1.47	-2.08	-2.64	$1.59E^{-04}$	$3.80E^{-06}$	$4.65E^{-08}$	0.36	0.24	0.16
SCLAV_3211	NLP/P60-family secreted protein	-1.01	-1.27	-2.01	$6.25E^{-04}$	$3.51E^{-05}$	$6.71E^{-08}$	0.50	0.41	0.25
SCLAV_3779	Lipoprotein	-1.25	-2.00	-2.03	$1.05E^{-03}$	$4.97E^{-06}$	$2.05E^{-06}$	0.42	0.25	0.24
SCLAV_3924	Putative secreted protein	-2.14	-2.99	-2.82	$1.94E^{-03}$	$3.97E^{-05}$	$5.56E^{-05}$	0.23	0.13	0.14
SCLAV_4356	Cell division-related protein	-1.37	-1.84	-1.96	$1.05E^{-04}$	$5.03E^{-06}$	$6.77E^{-07}$	0.39	0.28	0.26
SCLAV_4765	Amino-transferase	-2.76	-3.00	-3.46	$2.91E^{-04}$	$8.20E^{-05}$	$1.12E^{-05}$	0.15	0.12	0.09
SCLAV_4953	AmfT protein	-1.13	-2.11	-0.44	$1.30E^{-01}$	$3.60E^{-03}$	$5.88E^{-01}$	0.46	0.23	0.74
SCLAV_4954	Membrane translocator, AmfB	-0.34	-0.88	-0.25	$6.33E^{-01}$	$1.01E^{-01}$	$7.01E^{-01}$	0.79	0.54	0.84
SCLAV_4955	Membrane translocator, AmfA	-0.66	-1.56	0.33	$1.70E^{-01}$	$7.37E^{-04}$	$5.04E^{-01}$	0.63	0.34	1.26
SCLAV_4956	Transcriptional regulator, AmfR	-0.81	-1.28	-1.13	$9.28E^{-02}$	$4.92E^{-03}$	$9.98E^{-03}$	0.57	0.41	0.46

SCLAV_5096	Putative SsgD protein	-1.36	-1.23	-1.13	$1.93E^{-03}$	$3.34E^{-03}$	$5.42E^{-03}$	0.39	0.43	0.46
SCLAV_5177	Secreted protein	-3.40	-4.31	-6.18	$1.40E^{-03}$	$8.34E^{-05}$	$7.24E^{-07}$	0.10	0.05	0.01
SCLAV_5178	Hypothetical protein	-2.97	-3.71	-5.62	$3.26E^{-03}$	$2.71E^{-04}$	$1.46E^{-06}$	0.13	0.08	0.02
SCLAV_5179	Putative secreted protein	-2.59	-3.68	-4.77	$1.26E^{-03}$	$2.05E^{-05}$	$5.48E^{-07}$	0.17	0.08	0.04
SCLAV_5180	Putative secreted protein	-2.31	-2.80	-2.98	$5.33E^{-04}$	$4.34E^{-05}$	$1.48E^{-05}$	0.20	0.14	0.13
SCLAV_5181	Putative ABC transporter ATP-binding protein	-2.54	-2.57	-2.70	$8.28E^{-05}$	$5.17E^{-05}$	$2.01E^{-05}$	0.17	0.17	0.15
SCLAV_5182	Putative YrbE family protein	-2.12	-2.65	-1.54	$1.13E^{-02}$	$1.38E^{-03}$	$4.84E^{-02}$	0.23	0.16	0.34
SCLAV_5183	ABC-transporter integral membrane protein	-2.25	-2.84	-2.59	$6.82E^{-04}$	$3.71E^{-05}$	$7.47E^{-05}$	0.21	0.14	0.17
SCLAV_5184	Secreted protein	-2.44	-3.07	-2.14	$3.79E^{-05}$	$3.46E^{-06}$	$7.95E^{-05}$	0.18	0.12	0.23
SCLAV_5185	Putative Mce family protein	-2.64	-3.32	-3.47	$6.18E^{-04}$	$3.42E^{-05}$	$1.43E^{-05}$	0.16	0.10	0.09
SCLAV_5186	Secreted protein	-2.12	-2.76	-2.35	$3.32E^{-04}$	$1.20E^{-05}$	$5.20E^{-05}$	0.23	0.15	0.20
SCLAV_5187	Secreted protein	-2.60	-3.05	-3.04	$5.48E^{-05}$	$1.57E^{-05}$	$3.96E^{-06}$	0.17	0.12	0.12
SCLAV_5188	Secreted protein	-2.36	-2.82	-2.70	$5.22E^{-04}$	$5.08E^{-05}$	$6.08E^{-05}$	0.20	0.14	0.15
SCLAV_5189	Putative Mce family protein	-2.36	-3.11	-2.95	$2.32E^{-04}$	$7.51E^{-06}$	$8.87E^{-06}$	0.19	0.12	0.13
SCLAV_5192	Putative membrane protein	-2.75	-3.45	-3.55	$1.51E^{-04}$	$8.68E^{-06}$	$3.75E^{-06}$	0.15	0.09	0.09

Table S2. Oligonucleotides used in this work

Oligonucleotide Name	Sequence (5´to 3´)	Annealing Temperature (ºC)
**a) Used for microarrays validation by RT-qPCR**		
SCLAV_0014_F	CGGCTGTGGGAACTCGTC	62
SCLAV_0014_R	GTCGGGGTCGGGGTGGT	62
SCLAV_0785_F	CGGCAAGTCCTCCCTCAT	62
SCLAV_0785_R	ACAGGTTGAAGTGCTGGAAG	62
SCLAV_3410_F	GGCGGTGGAGCATGTGG	64
SCLAV_3410_R	GCGACCCAGAGGTGGAG	64
SCLAV_4035_F	GGAAGGGCAAGGACAAGAG	62
SCLAV_4035_R	GTGGCGAGGTAGGGGAAC	62
SCLAV_4464_F	CGCCGATGTCCTGCTGAC	66
SCLAV_4464_R	AGAACGGGGGTGTGCTC	66
SCLAV_4484_F	CGCACTTCGCCTACGAC	62
SCLAV_4484_R	GCGGATTCTCACGGTTGTTC	62
SCLAV_4650_F	CGGCATCGAGGAGATGAG	62
SCLAV_4650_R	AGGGGTAGAGGGTGGCGTA	62
SCLAV_5491_F	CGGCTACGACGATGTGC	64
SCLAV_5491_R	GAACCCCTTGGACACGAG	64
SCLAV_5580_F	GCAGCAGTGTGGAGTGGA	62
SCLAV_5580_R	GGCGGTTGACGGTGGTG	62
**b) Used for the analysis of the right chromosomal arm**		
SCLAV_5146_F	GCTGAAGAACCCCGAAGAC	58
SCLAV_5146_R	CGTGTTGACGCTGTTGGTG	58
SCLAV_5308_F	TTCGGAGAGCAGTTGGAGTT	64
SCLAV_5308_R	AACAGAGCAGCGGACCAG	64
SCLAV_5482_F	CCAGAGCCATCGGAGAAGAT	60
SCLAV_5482_R	GGACACCAGCCCCCAAT	60

SCLAV_5485_F	GCTGAACGGCTACGAGACC	60
SCLAV_5485_R	CAGATAGAGCGAGCAGAGTCC	60
SCLAV_5487_F	GAGAGCGGTGAGGGAGGA	60
SCLAV_5487_R	CTGGTGTGCTGCCCGTA	60
SCLAV_5491_F	CGGCTACGACGATGTGC	64
SCLAV_5491_R	GAACCCCTTGGACACGAG	64
SCLAV_5487_F*	GGTGTACGGGTGCTCGTC	60
SCLAV_5487_R*	CCTCCACGAGGAAGTGACC	60
SCLAV_5488_F	GAAAGAAAGATGCCGGACAC	64
SCLAV_5488_R	AGATCCAGGTCCTTGCACAT	66
SCLAV_5488_F*	ATGTGCAAGGACCTGGATCT	66
SCLAV_5490_R*	TGTCCCTTGGAGAGGATCAG	66
SCLAV_5489_F	AGGACCGTCGCGGTGAAC	68
SCLAV_5490_F	ATCTGCGCTACGACTTCGAC	61
SCLAV_5490_R	CTGTTCGGCGATGTAGCACT	61
SCLAV_5491_F*	TACTGCCCCTTCGACTATGC	62
SCLAV_5491_R*	GTTGGCGGTCAGATAGGAGA	62
SCLAV_5521_F	AGAGCTGGAGGCCGAAGT	60
SCLAV_5521_R	CCGGGTGAGGATGGTGAT	60
SCLAV_5580_F	GCAGCAGTGTGGAGTGGA	62
SCLAV_5580_R	GGCGGTTGACGGTGGTG	62
SCLAV_5585_F	TCAACGAGGTCTTCCTCACC	60
SCLAV_5585_R	CGTTCATCAGGGTGGTCCT	60
SCLAV_5692_F	CGCTGTACCTCACCCCATC	64
SCLAV_5692_R	GTGGCCGAGCAGGATCT	64
SCLAV_5719_F	CCAACACCGACCAGAACG	58
SCLAV_5719_R	CGAGGAAGGGCAAGCAGT	58
Right Telomere_F	CACTGCTTGCCCTTCCTC	64
Right Telomere_R	AGCCACATTTGCCCATACAC	64
